# Machine-learning-assisted material discovery of oxygen-rich highly porous carbon active materials for aqueous supercapacitors

**DOI:** 10.1038/s41467-023-40282-1

**Published:** 2023-08-01

**Authors:** Tao Wang, Runtong Pan, Murillo L. Martins, Jinlei Cui, Zhennan Huang, Bishnu P. Thapaliya, Chi-Linh Do-Thanh, Musen Zhou, Juntian Fan, Zhenzhen Yang, Miaofang Chi, Takeshi Kobayashi, Jianzhong Wu, Eugene Mamontov, Sheng Dai

**Affiliations:** 1 Chemical Sciences Division, Oak Ridge National Laboratory, Oak Ridge, TN 37831 USA; 2 Department of Chemistry, Institute for Advanced Materials and Manufacturing, University of Tennessee, Knoxville, TN 37996 USA; 3 Department of Chemical and Environmental Engineering, University of California, Riverside, 92521 CA USA; 4 Neutron Scattering Division, Oak Ridge National Laboratory, Oak Ridge, TN 37831 USA; 5 U.S. DOE Ames National Laboratory, Ames, IA 50011 USA; 6 Center for Nanophase Materials Sciences, Oak Ridge National Laboratory, Oak Ridge, TN 37831 USA; 7 Present Address: Department of Chemistry, Institute for Advanced Materials and Manufacturing, University of Tennessee, Knoxville, TN 37996 USA

**Keywords:** Synthetic chemistry methodology, Porous materials

## Abstract

Porous carbons are the active materials of choice for supercapacitor applications because of their power capability, long-term cycle stability, and wide operating temperatures. However, the development of carbon active materials with improved physicochemical and electrochemical properties is generally carried out via time-consuming and cost-ineffective experimental processes. In this regard, machine-learning technology provides a data-driven approach to examine previously reported research works to find the critical features for developing ideal carbon materials for supercapacitors. Here, we report the design of a machine-learning-derived activation strategy that uses sodium amide and cross-linked polymer precursors to synthesize highly porous carbons (i.e., with specific surface areas > 4000 m^2^/g). Tuning the pore size and oxygen content of the carbonaceous materials, we report a highly porous carbon-base electrode with 0.7 mg/cm^2^ of electrode mass loading that exhibits a high specific capacitance of 610 F/g in 1 M H_2_SO_4_. This result approaches the specific capacitance of a porous carbon electrode predicted by the machine learning approach. We also investigate the charge storage mechanism and electrolyte transport properties via step potential electrochemical spectroscopy and quasielastic neutron scattering measurements.

## Abstract (web summary)

Machine-learning technology provides a data-driven approach to find the critical features for ideal carbon-based supercapacitors. Here, the authors report machine-Learning assisted discovery of oxygen rich highly porous carbons that exhibits a high specific capacitance.

## Introduction

Aqueous supercapacitors are critical energy storage devices for applications that require high power density and long cycle lifetime, such as regenerative braking systems in electric vehicles, uninterruptible power supplies, and power levelers for electronics^1–4^. With the fast development of supercapacitors, diverse materials including porous carbons, metal oxides/carbides/nitrides, and conductive polymers have been optimized to pursue a higher energy density in supercapacitors, among which porous carbons are still the primary and widely used active materials for commercial aqueous supercapacitors^3,5–9^. The advantages of porous carbons for supercapacitors include power capability, long-term cycle stability, wide operating temperatures, and high Coulombic efficiencies^10–13^. The basic energy storage mechanism of carbon supercapacitors is through an electrical double-layer capacitance (EDLC), derived from the reversible charge separation at the interface of the electrolyte with the carbon surface^14,15^. The large surface area and appropriate pore structure of carbon supercapacitors are crucial to provide a large interfacial area for a large EDLC as well as a fast ionic mobility path for high charge/discharge rates^16–21^. However, the experimental exploration for appropriate pore structures necessitates the formulation of intricate synthetic strategies, and the execution of time-intensive experimental processes including tedious synthesis of various carbon materials, characterization of pore structures, electrochemical tests, and data analysis. All of which, in the absence of a definitive guideline, become a time-consuming endeavor^22–24^. Recently, the development of machine-learning technology provides a data-driven approach to learning from extensive previously reported works and points out the critical features of an ideal supercapacitor device with carbon-based electrodes^25,26^. The maximum value (250 F/g at a scan rate of 1 mV/s) for pristine carbon electrodes in 6 M KOH electrolyte has been predicted by the data-driven machine learning model based on the artificial neural network (ANN), which is achieved when the porous carbon has the specific surface areas of micropores (pores with widths not exceeding about 2 nm) and mesopores (pores with width between 2 and 50 nm) at 700 and 300 m^2^/g, respectively^26^. However, the low specific capacitance and bulk density of porous carbons limit their volumetric performance^27^. A heteroatom doped carbon surface with electro-active species such as N/O sites, and metal/metal oxide particles provides pseudocapacitance through quick and reversible faradic reactions, which contributes to high overall specific gravimetric and volumetric capacitance values^28–35^. Compared to the pristine carbon, the ANN predicted maximum value for N/O-doped carbons up to 570 F/g at a scan rate of 1 mV/s in base electrolyte (viz., 6 M KOH) occurs at the larger specific surface areas from micropores and mesopores (1400 and 1000 m^2^/g, respectively)^26,36^. The cyclic stability of carbon-based supercapacitors could be investigated by cycling or voltage holding tests^9^. According to machine learning aided cyclic stability prediction, enhanced cyclic stability could be achieved after complete activation of the electrodes and the construction of three dimensional porous structures^37^. Based on the guideline provided by ANN machine-learning results, the construction of highly porous carbon with appropriate pore structure and heteroatom doping is promising to achieve a high energy density for supercapacitors.

The theoretical upper limit of the specific surface area for carbonaceous materials was 2630 m^2^/g, which was calculated from infinite single-layer defectless graphene^38^. A higher surface area up to 7745 m^2^/g could be achieved by dividing the infinite layer into isolated six-membered rings, which maximizes the number of exposed ring faces and edges for graphitic carbons with sp^2^ hybridization^39^. Besides the graphitic carbons, the other way to expose more ring faces and edges is to avoid the alignment of six-membered rings by connecting six-membered rings with sp^3^-hybridized carbon or forming an amorphous structure^40,41^. For the synthesis of such a highly porous carbon as predicted by ANN, the choices of precursor material and activation strategy (the process of enhancing the surface area and porosity of carbon materials) should be carefully made, because they dominate the pore structure and functionality in the resultant carbons^42–45^. Among various carbon-rich precursors, hypercrosslinked polymers (HCPs: amorphous microporous three-dimensional networks based on covalent linkage of organic building blocks) stand out as emerging precursors for highly porous carbons due to their comprehensive advantages of adjustable chemical composition, mild operating condition (reaction temperature <100 °C under ambient pressure), low-cost reagents, and high yield^46,47^. As a large library of polymers, HCPs are generally synthesized by using a formaldehyde dimethyl ether cross-linker to knit cheap aromatic blocks through a simple one-step Friedel–Crafts reaction^48^. The fast kinetics of Friedel–Crafts reactions form strong linkages among aromatic blocks, resulting in a highly crosslinked porous network^49,50^. The diversity of building blocks, coupled with a versatile synthetic approach, gives HCPs great potential as precursors for highly porous carbons with various pore architectures and tailored functional groups^51–53^. On the other hand, the carbonization–activation strategy is an effective way to prepare highly porous carbons, in which the porous structures are mainly obtained by oxidation of carbonous precursors with physical activation agents (CO_2_, O_2_, air, or H_2_O) or chemical activation agents (KOH, Na_2_CO_3_, ZnCl_2_, or H_3_PO_4_) at high temperatures (above 800 °C)^54–57^. However, the synthesis of a hyperporous carbon with a specific surface area of more than 4000 m^2^/g is still challenging^58,59^. Besides, the high activation temperature above 800 °C in conventional strategies generally results in a serious loss of functional groups and a low carbon yield^53^. We previously reported the flux activation of mesoporous carbon or graphene in molten sodium amide (NaNH_2_) under a low activation temperature (230–380 °C), which can prepare nitrogen-doped carbons with a high carbon yield of up to 90%^60–62^. Thus, a combination of suitable HCPs and flux activation strategy was considered to achieve the target heteroatom doped hyperporous carbon for the ANN predicted high specific capacitance aqueous supercapacitor.

**Fig. 1:**
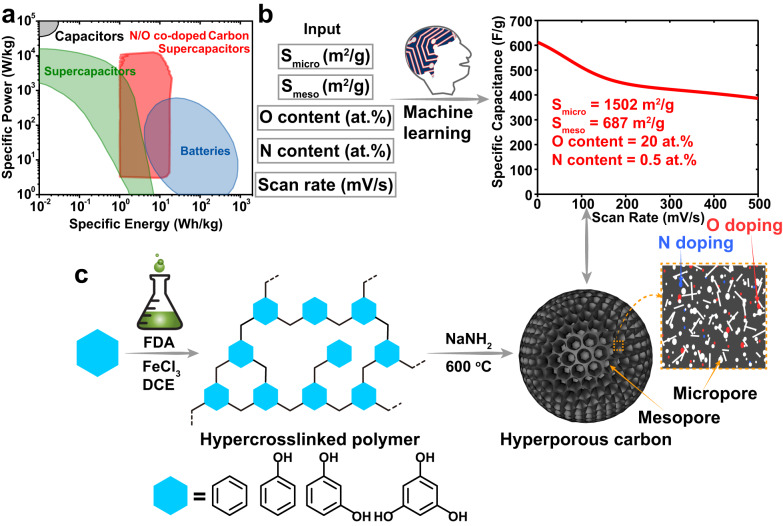
Machine learning guided synthesis of hyperporous carbons for supercapacitors. **a** Ragone plot for supercapacitors, batteries, and conventional electrical capacitors^26^. Copyright 2020 American Chemical Society. **b** Data structure and capacitance boundary predicted by the ANN model for N/O co-doped carbon electrodes in 1 M H_2_SO_4_ electrolyte. **c** Experimental design for the synthesis of the target hyperporous carbons predicted by the ANN model.

Herein, we extend our ANN model for the specific capacitance prediction (refers to active material only in this work) of N/O co-doped carbons in acid aqueous electrolyte solution using literature data collected in acid electrolyte, which pushes the specific capacitance up-limit of N/O co-doped carbons from 570 to 611 F/g (Fig. 1 a, b). The ANN predicted maximum value for N/O-doped carbons in 1 M H_2_SO_4_ occurs at the specific micropore surface area of 1502 m^2^/g and mesopore surface area of 687 m^2^/g, together with the O content of 20 atom% and the N content of 0.5 atom%. Based on the machine learning result and previous works about the design of hyperporous carbonaceous materials, we designed a series of experiments to achieve hyperporous carbons with high surface areas over 4000 m^2^/g, O content over 10 wt%, and N content around 1 wt%. HCPs as the precursors for hyperporous carbons were synthesized by binding benzene or phenols (phenol, resorcinol, or phloroglucinol) with dimethoxymethane (FDA) through a one-step Friedel–Crafts reaction (Fig. 1c). Hyperporous carbons were obtained by carbonization of HCPs under 600 °C using NaNH_2_ as an activation agent, in which the low carbonization/activation temperature was used to retain a high heteroatom doping content in products. Hyperporous carbons using different HCP precursors were named after their corresponding monomers. The Brunauer-Emmett-Teller (BET) surface area of hyperporous carbons increases with the number of phenolic hydroxyl groups, from 2903 m^2^/g of benzene-based hyperporous carbon (C-Ben) to 4476 m^2^/g of phloroglucinol based hyperporous carbon (C-Phl). In agreement with the ANN prediction, the specific capacitance of C-Phl is as high as 610 F/g at a scan rate of 1 mV/s and 628 F/g at a specific current of 0.2 A/g in 1 M H_2_SO_4_ solution. These hyperporous carbons with high surface area, optimized micro/meso pore structure, and abundant N/O doping can approach the capacitance boundary of carbon active materials for aqueous carbon supercapacitor applications. The capacitance contributions from pores and heteroatoms were investigated by step potential electrochemical spectroscopy (SPECS) and quasielastic neutron scattering (QENS) experiments, where mesopores, especially heteroatom-doped ones, contributed the most to overall capacitance.

## Results

### ANN machine learning

ANN was trained based on the data collected from the literature (Supplementary Table 1). The structural features used in ANN include micropore surface area (S_micro_) and mesopore surface area (S_meso_), while the total percentage of nitrogen and oxygen doping is used as the chemical features (Supplementary Note 1). In addition, the scan rate of cyclic voltammetry is also used to capture the decrease of specific capacitance at a faster charging/discharging rate. In the training database, the electrochemical performance of N/O co-doped activated carbon-based electrodes are collected in both 6 M KOH and 1 M H_2_SO_4_ electrolyte. In the ANN model, the type of electrolyte is treated as a dummy variable. The size of the dataset is 288 data points. The percentages of training, validation, and test dataset are 70%, 15%, and 15%, respectively. The ANN includes a layer of 7 neurons as a hidden layer with a hyperbolic tangent sigmoid transfer function. The backpropagation employs Bayesian regularization, which makes the ANN more robust and more generalized without overfitting. As shown in Supplementary Fig. 1, the best training performance was achieved at epoch 35, where the mean square error (MSE) for the validation dataset was 1189. As shown in Supplementary Fig. 2, ANN can correlate the electrochemical performance well over a wide range of specific capacitance with root mean square errors (RMSE) of 25.0 for the training dataset, 34.5 for the validation dataset, and 38.5 for the test dataset. The color mapping of specific capacitance vs. O/N content at 5 mV/s reveals a clear increase in specific capacitance with O content (Supplementary Fig. 3a). 20 at% was set as a boundary of O content because no higher value has been reported for carbon-based supercapacitors. The color mapping of specific capacitance vs. S_meso_/S_mico_ content at 5 mV/s indicates a maximum specific capacitance value when the specific surface areas from micropores and mesopores are 1502 and 687 m^2^/g, respectively (Supplementary Fig. 3b). Based on literature data, ANN predicts the maximum capacitance of N/O co-doped activated carbon electrode in 1 M H_2_SO_4_ can be achieved with the micropore surface area of 1502 m^2^/g, mesopore surface area of 687 m^2^/g, nitrogen-doping of 0.5 at% and oxygen-doping of 20 at%. Figure 1b shows the predicted specific capacitance and retention versus scan rate for the best N/O co-doped activated carbon electrode. According to the ANN prediction, in 1 M H_2_SO_4_ electrolyte, excess oxygen doping would lead to a significant increase in specific capacitance due to the increase of electronic conductivity and improvement of the electrode surface wetting.

### Synthesis and activation of phloroglucinol based HCP

**Fig. 2:**
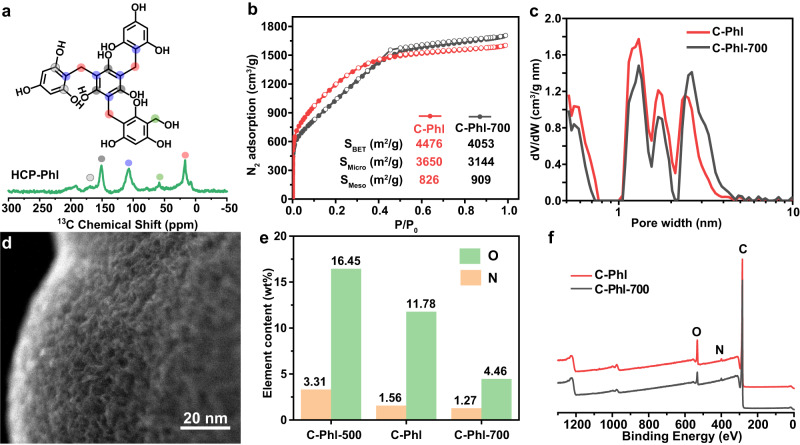
Physicochemical characterizations of HCP-Phl, C-Phl, and C-Phl-700. **a** ^13^C{^1^H} CP/MAS NMR of HCP-Phl. **b** N_2_ adsorption-desorption isotherms of C-Phl and C-Phl-700 at 77 K. **c** NLDFT pore distributions of C-Phl and C-Phl-700. **d** STEM image of C-Phl. **e** O and N contents in C-Phl-500, C-Phl, and C-Phl-700 calculated from element analysis. **f** XPS spectra of C-Phl and C-Phl-700.

Phloroglucinol units were hypercrosslinked via the Friedel–Crafts reaction^48^, and the resulting polymers are named HCP-Phl. During the crosslinking process, FDA was used as an external linker to knit the aromatic ring in phloroglucinol with methylene groups. The formation of methylene groups is validated by the strong resonance peak at 17.5 ppm in ^13^C{^1^H} cross-polarization magic-angle spinning (CP/MAS) NMR spectrum (Fig. 2a). A weak signal at 58 ppm is attributed to a methoxy group attached to aromatic carbons. The strong resonance peak at 107 ppm is assigned to aromatic carbons bonded to methylene linkers, and the peaks at 150–160 ppm are assigned to aromatic carbons connected to OH. The NMR results validate the successful crosslinking of phloroglucinol with methylene groups. HCP-Phl as the carbon precursor was mixed with NaNH_2_ and heated to 350 °C (N_2_ atmosphere) to pre-activate polymer in molten NaNH_2_. Then the mixture was further heated to 600 °C (N_2_ atmosphere) to obtain hyperporous carbon (C-Phl). C-Phl-700 was synthesized as a reference sample by changing the activation temperature from 600 °C to 700 °C. The pore structures and surface areas of hyperporous carbons were investigated by N_2_ adsorption-desorption isotherms at 77 K. As shown in Fig. 2b, C-Phl and C-Phl-700 having similar N_2_ adsorptions, are both hyperporous carbons with high BET surface areas (S_BET_s) of 4476 and 4053 m^2^/g, respectively. The detailed calculation process of S_BET_ is in the supplementary information (Supplementary Fig. 4–5 and Supplementary Note 2). Though the S_BET_ of C-Phl-700 is lower than that of C-Phl, C-Phl-700 has a slightly larger surface area from mesopores, corresponding to more abundant and larger mesopores in the non-local density functional theory (NLDFT) pore distribution (Fig. 2c). Mesopores with widths from 2 to 5 nm in C-Phl can be directly observed in the scanning transmission electron microscopy (STEM) image (Fig. 2d), which is consistent with the NLDFT pore distribution. Besides, the low-temperature activation leads to retaining a high oxygen content of 11.78 wt% in C-Phl (Fig. 2e). The increase of activation temperature from 600 °C to 700 °C results in a decreased O content from 11.78 to 4.46 wt%. The XPS spectra of C-Phl and C-Phl-700 reveal the signals from C, N, and O 1 s spectrum (Fig. 2f), indicating the O/N co-doping of the carbon. The C 1 *s* spectra show the C sp^2^, C sp^3^, C sp^3^-OH, and C sp^2^ = O peaks at 284.5, 285.2, 285.8, and 286.3 eV, respectively, where the sp^3^ C is 20% in total carbon (Supplementary Fig. 6)^63^. The absence of peaks in the XRD pattern (Supplementary Fig. 7) and the high *I*_D_/*I*_G_ values above 3 in Raman spectra (Supplementary Fig. 8) validate the amorphous nature of C-Phl and C-Phl-700. Compared to the target structure predicted by ANN machine learning (S_micro_ = 3900 m^2^/g, S_meso_ = 1000 m^2^/g), C-Phl has a similar S_micro_ of 3650 m^2^/g and S_meso_ of 826 m^2^/g. Moreover, the low-temperature NaNH_2_ activation can achieve high oxygen contents above 10 wt% and nitrogen doping around 1 wt% in hyperporous carbons. Thus, C-Phl and C-Phl-700 are hyperporous carbons with abundant heteroatom doping, which provides a route to approach the predicted capacitance boundary of porous carbons.

### Electrochemical characterizations of the hyperporous carbons

**Fig. 3:**
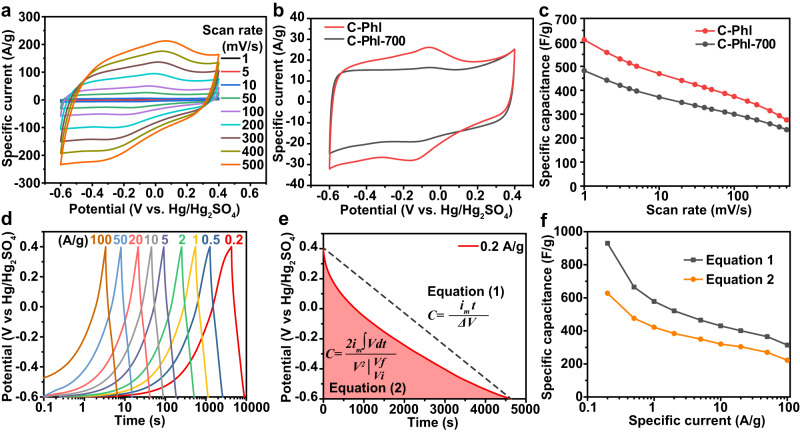
Electrochemical characterizations of C-Phl and C-Phl-700 using three-electrode cells at 20 °C. The mass loading of the working electrode is 0.7 mg/cm^2^. **a** CV curves of C-Phl at different scan rates from 1 to 500 mV/s. **b** CV curves of C-Phl and C-Phl-700 at 50 mV/s. **c** Specific capacitances at different scan rates calculated from CV curves. **d** GCD curves of C-Phl at different specific currents from 0.2 to 100 A/g. **e** Discharge curve of C-Phl at 0.2 A/g. **f** Specific capacitances of C-Phl at different specific currents calculated from Eqs. (1) and (2).

The electrochemical energy storage properties of hyperporous carbons were initially evaluated by cyclic voltammetry (CV) measurements at different scan rates from 1 to 500 mV/s in a conventional three-electrode system with 1 M H_2_SO_4_ solution as the electrolyte (Fig. 3a). The hyperporous carbons exhibited high specific capacitance values up to 610 F/g at 1 mV/s, which surpasses the ANN model-predicted highest specific capacitance value (570 F/g) of porous carbons in 6 M KOH and approaches the specific capacitance boundary of porous carbon in 1 M H_2_SO_4_. The similar pore structures and different O contents of C-Phl and C-Phl-700 make them good models for investigating the influences of pore structure and O doping on the electrochemical energy storage properties of hyperporous carbons. As shown in Fig. 3b, C-Phl and C-Phl-700 both exhibited rectangular box-shaped cyclic voltammograms, while the enclosed area of the CV curve was larger in C-Phl. Besides the stretched rectangle shape, the CV curves of hyperporous carbons reveal peaks due to the redox processes of N/O-species and broad distributions of peaks due to delocalized electrons. The larger specific capacitance of C-Phl is mainly from its redox peaks around −0.1 V (vs. Hg/Hg_2_SO_4_), benefiting from its higher O contents. The specific capacitance of C-Phl is higher than that of C-Phl-700 at 1 mV/s (610 vs. 482 F/g, Fig. 3c), while the gap narrows at higher scan rates (e.g., 277 vs. 236 F/g at 500 mV/s), indicating a better rate performance of C-Phl-700. The rate performance of C-Phl was further investigated by galvanostatic charge-discharge (GCD) tests under different specific currents from 0.2 to 100 A/g (Fig. 3d). According to the previous literature, the average specific capacitance based on GCD curves was initially calculated to be 930 F/g at a specific current of 0.2 A/g by using Eq. (1):

1 $$C=i_{m}t/\varDelta V$$

where *i*_*m*_ (A/g) is the specific discharge current, *ΔV* is the difference in discharge voltage, and *t* is the discharge time (Fig. 3e). However, the discharge curve of C-Phl exhibited a negative slope, which means the faradic reaction charge/voltage ratio does not remain constant but varies with time. The nonlinearities in GCD curves are characteristic behavior of porous electrode capacitors, which cannot be properly described by a classic *RC* model^64^. Thus, the use of Eq. (1) will overestimate the capacitance from GCD curves. For this case, the integration of Eq. (2) was suggested to describe the average capacitance of non-symmetric discharge curves^65^:

2 $$C=\frac{2{i}_{m}\int {Vdt}}{{V}^{2}{{{{{{\rm{|}}}}}}}_{{Vi}}^{{Vf}}}$$

where *i*_*m*_ (A/g) is the specific discharge current, $\int {Vdt}$ is the integral area of the discharge curve, *V*_*i*_ and *V*_*f*_ are the initial and final voltages, respectively. As shown in Fig. 3f, the specific discharge capacitance was calculated as 628 F/g at 0.2 A/g using Eq. (2), which was close to the value (610 F/g) calculated from CV curves at 1 mV/s. In addition, the specific capacitance of C-Phl was as high as 223 F/g even at a high specific current of 100 A/g, suggesting good rate performance. Indeed, the specific capacitance of C-Phl remained 89% after 10,000 GCD cycles at a specific current of 20 A/g (Supplementary Fig. 9) and 92% after 25,000 cycles at a higher specific current of 50 A/g (Supplementary Fig. 10–11). During the voltage hold test, a constant potential of 0.4 V was applied to the working electrode and 4 GCD cycles were performed after every 10 h of voltage holding. A capacitance loss of 47% occurs after 500 h of the voltage hold test to validate that the constant voltage hold test is more time efficient than a GCD cycling test (Supplementary Fig. 12). Electrochemical impedance spectroscopy (EIS) was used to investigate the impedance of hyperporous carbon electrodes. The overall Nyquist plots of the C-Phl and C-Phl-700 almost coincide, suggesting their similar resistance behaviors (Supplementary Fig. 13a). At low frequencies from 0.1 to 1 Hz, the plots of C-Phl and C-Phl-700 dispersed in nearly vertical lines, which were close to that of an ideal capacitor. At middle frequencies from 10 to 100 Hz, the Nyquist points have a slope of 45° because of the distributed resistance of porous carbon electrodes^66^. At high frequencies above 100 Hz, the electric series resistance (ESR) is higher in C-Phl than that in C-Phl-700, which includes the electrolyte resistance, electrode/electrolyte interface resistance, and the current collector/electrode contact resistance (Supplementary Fig. 13b)^67^. Since the electrolyte and current collector are the same for different tests, the higher ESR indicates a slightly lower electronic conductivity of C-Phl than that of C-Phl-700. Besides the low loading test (0.7 mg/cm^2^ of active material), a high mass loading of 4.2 mg/cm^2^ was used to evaluate the high-loading performance of C-Phl. As shown in Supplementary Fig. 14, the specific capacitances of high-loading electrodes at a low scan rate below 10 mV/s is very close to those of low loading ones. The volumetric capacitance of high loading electrodes at 1 mV/s is 776 F/cm^3^, higher than that of low loading ones (712 F/cm^3^, Supplementary Fig. 15). The rate performance of high loading electrodes at higher scan rates above 20 mV/s is not as good as that of low loading ones, mainly due to its increased electrode thickness from 30 to 157 μm.

### Understanding the charge storage mechanism in hyperporous carbons

**Fig. 4:**
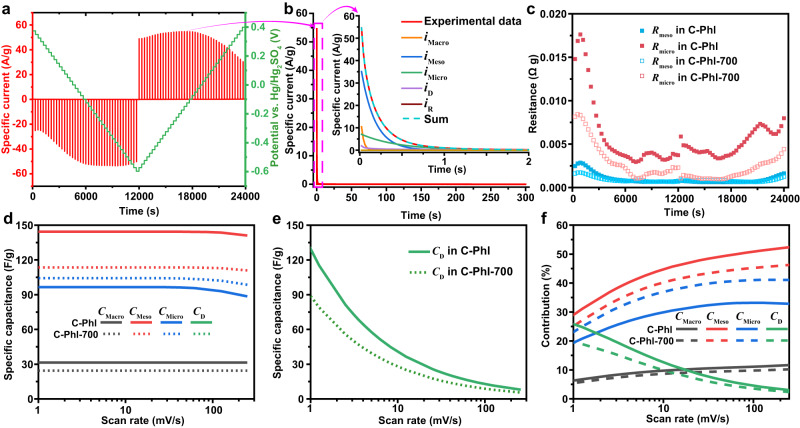
SPECS analysis for C-Phl and C-Phl-700 using three-electrode cells at 20 °C. The mass loading of the working electrode is 0.7 mg/cm^2^. **a** SPECS data of C-Phl collected in 1 M H_2_SO_4_. **b** An individual current response deconvoluted in *i*_macro_, *i*_meso_, *i*_micro_, *i*_D_, and *i*_R_. **c** Different resistances (*R*_Micro_ and *R*_Meso_) in C-Phl and C-Phl-700 fitted from the SPECS data. **d** Breakdown capacitances (*C*_Micro_, *C*_Meso_, *C*_Macro_) in C-Phl and C-Phl-700 calculated from the SPECS data. **e** Capacitances contributed from diffusion processes (*C*_D_) in C-Phl and C-Phl-700 calculated from the SPECS data. **f** Comparison of capacitance contributions in C-Phl and C-Phl-700.

To gain insight into the energy storage mechanism of hyperporous carbons, we previously developed a modified step potential electrochemical spectroscopy (SPECS) to distinguish the detailed capacitance contributions from pores and redox processes^16^. Here, the SPECS method was used to distinguish the capacitance contributions from micro/mesopores and diffusion-limited redox processes in hyperporous carbons (Fig. 4). SPECS experiments were performed by applying staircase voltammetry with a potential step of 25 mV and an equilibration time of 300 s on the working electrode (Fig. 4a). The current response as a function of time is described by three double-layer charging processes from geometric surface/macropores, mesopores, micropores (noted as *i*_Macro_, *i*_Meso_, and *i*_Micro_, respectively), a diffusion-limited process (*i*_D_), and a residual equilibrium current (*i*_R_) using the Eq. (3):

3 $$\begin{array}{c}i(t)=i{{{{{\mathrm{Macro}}}}}}+i{{{{{\mathrm{Meso}}}}}}+i{{{{{\mathrm{Micro}}}}}}+i_{D}+i_{R}\\=\frac{\varDelta E}{{R}_{{{{{{\rm{Macro}}}}}}}}\exp \left(-\frac{t}{{R}_{{{{{{\rm{Macro}}}}}}}{C}_{1}}\right)+\frac{\varDelta E}{{R}_{{{{{{\rm{Meso}}}}}}}}\exp \left(-\frac{t}{{R}_{{{{{{\rm{Meso}}}}}}}{C}_{2}}\right)+\frac{\varDelta E}{{R}_{{{{{{\rm{Micro}}}}}}}}\exp \left(-\frac{t}{{R}_{{{{{{\rm{Micro}}}}}}}{C}_{3}}\right)+\frac{B}{{t}^{1/2}}+{i}_{R}\end{array}$$

where *C*_*1*_, *C*_*2*_, *C*_*3*_, *R*_Macro_, *R*_Meso_, *R*_Micro_, *B*, and *i*_R_ are constants obtained by the nonlinear fitting. The well-matched experimental data and the sum of the fitting currents (Fig. 4b) indicate the good modeling of the current response by Eq. (3). The electric double-layer formation is differentiated on different surfaces due to their different time constants (*τ* = *RC*; s). As shown in Fig. 4c and Supplementary Fig. 16, the resistance and time constant in micropores (*R*_micro_ and *τ*_Micro_) are larger than those in mesopores (*R*_meso_ and *τ*_Meso_), respectively. The subdivided current responses were converted to voltammograms to calculate the breakdown pore capacitances. Micropores are more effective than mesopores in contributing a high surface area under a certain pore volume. However, due to the small pore size of micropores, the diffusion and packing density of ions are limited, which result in a lower charge storage density compared to that in mesopores^16,68–70^. Thus, the mesopores rather than micropores contributed the most to capacitive capacitance in both C-Phl and C-Phl-700 (Fig. 4d), though the surface areas of these hyperporous carbons are mainly contributed from micropores. The micropore capacitance contributions, especially the one of C-Phl, decrease fast at high scan rates above 100 mV s^−1^, resulting in capacitance loss under high scan rates. While the capacitance loss at low scan rates (<10 mV s^−1^) is mainly from the decrease of capacitance from diffusion-limited redox processes (Fig. 4e). Compared to C-Phl-700, C-Phl exhibited higher overall capacitance because of its higher capacitance contributions from mesopores and diffusion-limited redox processes (Fig. 4f). It is easy to understand that the higher pseudocapacitance from the diffusion-limited redox process in C-Phl is due to the additional O/N sites. However, further investigation is needed to explain why C-Phl having a lower mesopore surface area than C-Phl-700 exhibited higher capacitance from mesopores.

### Proton transport in hyperporous carbons

**Fig. 5:**
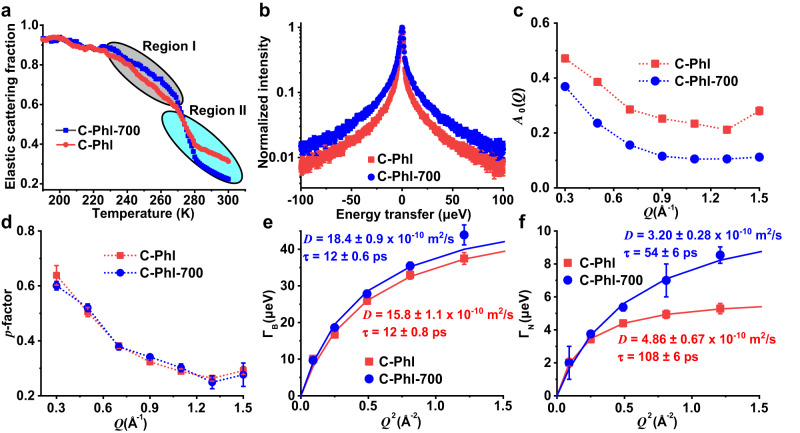
Quasielastic neutron scattering analysis. **a** Elastic scattering intensity scans for the wet samples normalized by the samples’ mass and with the datapoints at *T* = 20 K normalized to unity. The data is summed over *Q* = 0.3–1.1 Å^−1^. **b** QENS spectra collected at 300 K presented as the dynamic scattering functions normalized to unity. *Q*-dependences of ${A}_{0}$(*Q*) (**c**) and *p* (**d**) as obtained by fitting the QENS data with Eqs. (4) and (5). *Q*^2^-dependences of $\Gamma$_B_ (*Q*) (**e**) and $\Gamma$_N_ (*Q*) (**f**) presented together with the correspondent fits with Eq. (6). The values obtained for D and *τ*_0_ are also shown in the figure. In (**a**) and (**b**), the error bars (mostly not visible since these are within the same dimensions of the symbols) depict the experimental uncertainty as defined by Poisson statistics, $\delta \left(N\right)=\,\sqrt{N}$, where *N* is the number of counts in a given frequency bin and, therefore, the fractional error is $\frac{\delta (N)}{N}=\frac{1}{\sqrt{N}}$. In (**c**–**f**), the error bars indicate the standard deviations of the parameters determined by fitting the data with Eqs. (4–6).

The QENS experiments were performed to achieve a quantitative description of the proton transport properties in the hyperporous carbons (C-Phl and C-Phl-700). The experiments were conducted at the backscattering silicon (BASIS) spectrometer^71^, which provides an energy resolution of 3.7 μeV (full-width at half-maximum for the *Q*-averaged resolution value) and an accessible dynamical range of ±100 μeV. Hyperporous carbons were soaked overnight in 1 M H_2_SO_4_ and separated by fast vacuum filtration to make the wet samples. First, temperature-dependent measurements of the elastic scattering intensities were performed (Supplementary Fig. 17a, b). For the wet samples, both C-Phl and C-Phl-700 present a first detectable activation of dynamic events around 230 K (Region I in Fig. 5a) and a second activation around 273 K (Region II in Fig. 5a). While the first may be related to the electrolyte within the micropore structure of the carbon and/or with protons involved in ionic solvation, the latter resembles the behavior expected for bulk-like water or aqueous solutions and is related to motions of an independent population of the electrolyte within mesopores. C-Phl-700 presents a lower percent of elastic scattering, 12%, than C-Phl, 20% at 300 K (Supplementary Fig. 17c). Then, full QENS spectra were collected at 300 K and are presented in Fig. 5b at Q = 0.7 Å^−1^, Supplementary Fig. 18 and 19. In Fig. 5b, the data are normalized to unity to allow for visual observation of the faster relaxations in C-Phl-700 as compared with those in C-Phl, as depicted by the sharper signal of the latter. For a quantitative description of the QENS signals collected at 300 K, they were fitted with the following expression:

4 $$I\left(Q,\, E\right)=\left[{A}_{0}\left(Q\right)\delta \left(E\right)+\left(1-{A}_{0}\left(Q\right)\right)S\left(Q,\, E\right)\right]\otimes R\left(Q,\, E\right)+B\left(Q,\, E\right)\,$$

where *A*_*0*_(*Q*) is the fraction of elastic scattering, *δ*(*E*) is a Dirac delta function that accounts for the elastic scattering (zero energy transfer), *S*(*Q, E*) is the model dynamic scattering function, *R*(*Q, E*) is the instrument resolution function, and *B*(*Q, E*) is a linear background. For the dry samples, *A*_*0*_(*Q*) = 1, that is, no dynamic events could be detected within the instrumental resolution of BASIS. For the wet samples, *S*(*Q, E*) has been described as a sum of two Lorentzian functions:

5 $$S\left(Q,\, E\right)=p\left[\frac{1}{\pi }\frac{{\Gamma }_{N}(Q)}{{{\Gamma }_{N}}^{2}\left(Q\right)+\,{E}^{2}}\right]+(1-p)\left[\frac{1}{\pi }\frac{{\Gamma }_{B}(Q)}{{{\Gamma }_{B}}^{2}\left(Q\right)+\,{E}^{2}}\right]$$

where *p* refers to the spectral weight of slow (narrow component) dynamics to the QENS signal, $\Gamma$_*N*_(*Q*) is the half-width at the half maximum of the narrow component, and $\Gamma$_*B*_(*Q*) is the half-width at the half maximum of a broader (faster dynamics) component. The *Q*-dependence of the broadening of the QENS Lorentzian components were then fitted with a jump-diffusion model:

6 $$\Gamma (Q)=\frac{\hslash D{Q}^{2}}{1+D{Q}^{2}{\tau }_{0}}$$

where *τ*_0_ is the residence time between jumps amid two sites and *D* is the diffusion coefficient. With these parameters, the average jump length (*L*) can also be obtained by $L=\,\sqrt{6D{\tau }_{0}}$. The behaviors of *A*_*0*_(*Q*) and the *p*-factor are presented in Fig. 5 c and d as functions of Q. As for *A*_*0*_(*Q*), in Fig. 5c, the outcomes are nearly the same as the ones previously displayed in Supplementary Fig. 17c and Supplementary Note 3, that is, C-Phl retains a larger content of electrolyte immobilized on the carbon walls. Regarding the p-factor, Fig. 5d, it is identical for C-Phl −700 and C-Phl, exposing that even if C-Phl-700 accommodates a larger content of electrolyte within its structure, this exceeding volume does not disturb the balance between slow and fast relaxations. In both cases, the slow dynamics are responsible for ~30% of the motions within the QENS signal.

In Fig. 5e and f, the *Q*^2^-dependences of $\Gamma$_N_(*Q*) and $\Gamma$_B_(*Q*) are presented together with the correspondent fits with Eq. (6) and the values obtained for *D* and *τ*_0_. As presented in Fig. 5e, C-Phl-700 presents a higher diffusion coefficient for the broad component (18.4 ± 0.9 × 10^−10^ m^2^/s versus 15.8 ± 1.1 × 10^−10^ m^2^/s in C-Phl) but the relaxation times are identical in both samples. Here, the diffusion coefficients are lower than the value of the diffusion coefficient of bulk water (~23 × 10^−10^ m^2^/s)^72^ and comparable to the diffusivity previously determined for a 3.2 M solution of H_2_SO_4_ at 290 K (~12 × 10^−10^ m^2^/s)^73^. As for the relaxation times of the fast relaxations, these are considerably higher than the values previously obtained for water and H_2_SO_4_ solutions (~1 and ~3 ps), and, from the values of *D* and *τ*_0_, the jump lengths have been determined as 3.6 ± 0.2 Å and 3.4 ± 0.3 Å for C-Phl-700 and C-Phl, respectively. These values are about twice the value expected for bulk water at 293 K, which is <2 Å^72^ and considerably higher than the value once determined for a 3.2 M solution of H_2_SO_4_ at 290 K, 0.6 Å^73^. Since the loosely bound water molecules in the outer surfaces of carbon were removed as well as those in macropores, and both hyperporous carbons exhibited micropores and mesopores in NFT pore distribution (Fig. 2c), the broad components in the QENS signals should originate from bulk-like populations in mesopores. The higher diffusion coefficient for the broad component in C-Phl-700 validates the faster diffusion of electrolyte in C-Phl-700 than that in C-Phl. Turning to $\Gamma$_N_(*Q*), whose *Q*^2^-dependences are presented in Fig. 5f, clearer differences can be observed between C-Phl-700 and C-Phl. Here, despite the lower diffusion coefficient of C-Phl-700 as compared with C-Phl determined by fitting the data with Eq. (6), the large error bar in the first data point of C-Phl-700 does not allow for affirming that such a difference is indeed considerable. Regardless, C-Phl-700 does exhibit a shorter relaxation time, which is consistent with the larger amount of electrolyte that this sample accommodates, as discussed in previous work with carbon-based matrixes with different levels of hydration^74^. For the narrow component, the jump lengths have been determined as 3.2 ± 0.5 Å and 5.6 ± 0.2 Å for C-Phl-700 and C-Phl, respectively. Hence, C-Phl-700 presents nearly the same jump length in both the fast and slow relaxations, while C-Phl has a larger jump length in the slow dynamics.

Overall, the QENS results indicate that both samples do have very similar microstructures and, once into the matrixes, the electrolyte is separated into two independent fractions: a bulk-like mesopore-resident population and a micropores-resident population. These generate fast and slow QENS components, respectively. Despite the larger amount of electrolyte within C-Phl-700, there is no difference in the balance between these populations, as, in both samples, the dynamics from the micropores-resident electrolyte accounts for ~30% of the total scattering. There are, however, differences in the dynamics of the electrolytes within the carbons as both the slow and the fast components are faster in C-Phl-700 as compared with C-Phl. C-Phl immobilizes a larger content of the electrolyte on the carbon walls. Based on the element analysis in Fig. 2e, the N contents were similar in C-Phl and C-Phl-700 (1.56 wt% vs. 1.27 wt%), while the O content in C-Phl was much higher than that of C-Phl-700 (11.78 wt% vs. 4.46 wt%). The major difference in functional groups between C-Phl and C-Phl-700 is from O-containing groups. A higher O content in C-Phl (Fig. 2e) could be responsible for the enhanced electrolyte attraction to the carbon, similar to the earlier observation made in QENS studies of carbon materials^75^. To identify the detailed functional groups, the XPS O 1 s peaks of both C-Phl and C-Phl-700 were fitted by two peaks from HO-C and O = C groups, in which HO-C groups occupy more than 90% of O-containing groups based on their peak area ratios (Supplementary Fig. 20). Thus, the interaction between HO-C group and electrolyte might be the main reason for the enhanced electrolyte attraction to carbon surface. The hydrogen bonding between hydroxyl groups and water can insert into the outer sheath of hydronium ions to interrupt the hydrogen bonds of water molecules with decreased water activity^76,77^. The high attraction efficiency of the electrolyte components into the pores and onto the carbon surfaces can draw ions closer to pore surfaces and enhance potential-driven ion transport during electrosorption. On this basis, the functional groups in C-Phl are more efficient in attracting the electrolyte components into the pores and onto the carbon surfaces, which may be a decisive factor for the higher capacitance in this sample. The finding about a bulk-like mesopore resident population and a micropores-resident population explained the high-rate performance of C-Phl-700, which exhibited the faster dynamics of the electrolytes within the carbons as both the slow and the fast components compared to C-Phl. As for the design of electrolyte, a stronger interaction between electrolyte and carbon is conducive to a larger capacitance but a lower rate performance due to the slower dynamics of the electrolyte. So, a balance between energy density and power density can be achieved by surface modification of carbon or choice of electrolyte.

### General synthesis of hyperporous carbons

To prove the general activation method for the synthesis of hyperporous carbons, benzene, phenol, and resorcinol were hypercrosslinked using a similar process as the synthesis of HCP-Phl^48^, and the resulting polymers are referred to as HCP-Ben, HCP-Phe, and HCP-Res, respectively. As shown in Supplementary Fig. 21, the resonance peaks near 137 and 130 ppm in the ^13^C NMR of HCP-Ben originated from substituted and unsubstituted aromatic carbons, respectively. Insertion of methylene linkers at m-positions in phenol should result in the formation of aromatic carbon resonating at 135–140 ppm, which is not found in the ^13^C CP/MAS NMR of HCP-Phe. The absence of that signal suggests that the aromatic rings are linked via ortho and/or meta-positions in HCP-Phe, corresponding to the resonance peak at 130 ppm. The sharp peak at 150 ppm corresponds to aromatic carbon bonding to the hydroxyl group, indicating that phenolic hydroxyl groups are intact after cross-linking. All the ^13^C NMR spectra of HCPs reveal peaks corresponding to methylene groups (15–35 ppm), and the peak position shifts to the higher field side with the increase of hydroxyl groups in the monomer. The peaks of methylene linkers in NMR validate the successful synthesis of HCPs based on benzene, phenol, resorcinol, and phloroglucinol.

**Fig. 6:**
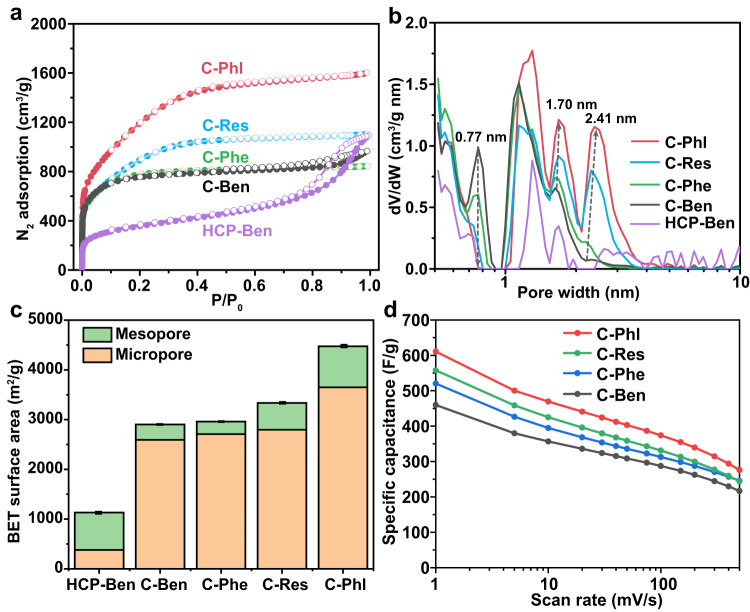
Physicochemical and electrochemical characterizations of hyperporous carbons from different HCP precursors. **a** N_2_ adsorption–desorption isotherms of hyperporous carbons and HCP-Ben at 77 K. **b** NLDFT pore distributions of hyperporous carbons and HCP-Ben. **c** BET surface areas with contributions from micropores and mesopores of hyperporous carbons and HCP-Ben. Error bars are standard deviations for BET surface areas. **d** Specific capacitances of hyperporous carbons at different scan rates calculated from CV curves using three-electrode cells at 20 °C. The mass loading of the working electrode is 0.7 mg/cm^2^.

After NaNH_2_ activation at 600 °C, C-Ben exhibited a much higher N_2_ uptake at a low-pressure region below 0.1 than HCP-Ben, indicating the formation of micropores during the activation process (Fig. 6a). The detailed pore distribution was calculated based on the NLDFT model. As shown in Fig. 6b, the pore distribution of C-Ben exhibited significantly increased peaks below 2 nm compared to that of HCP-Ben, corresponding to the larger surface area contributed by micropores in C-Ben. Meanwhile, the BET surface area (S_BET_) increased from 1132 m^2^/g of HCP-Ben to 2903 m^2^/g of C-Ben (Fig. 6c). The N_2_ adsorption isotherm of C-Phe is almost coinciding with that of C-Ben, despite the slightly higher N_2_ adsorption in C-Ben at the high-pressure region above 0.9. Considering the much lower BET surface area of HCP-Phe than that of HCP-Ben (16 vs. 1132 m^2^/g), the similar S_BET_ of C-Phe and C-Ben (2961 vs. 2903 m^2^/g) indicates that the introduction of phenolic hydroxyl groups contributes to a larger S_BET_ increment during NaNH_2_ activation process. HCP-Res and HCP-Phl, having more phenolic hydroxyl groups, exhibited higher S_BET_s of 3336 and 4476 m^2^/g in their carbon products C-Res and C-Phl, respectively (Fig. 6c). According to their pore distributions, the increase of phenolic hydroxyl groups in HCPs helps to achieve more micropores above 1.5 nm and mesopores below 3 nm but fewer micropores around 0.8 nm in hyperporous carbons (Fig. 6b). Besides the contribution to the high overall surface area, changing the monomer from benzene to phloroglucinol, the mesopore surface area (S_meso_) of the hyperporous carbon product increases from 310 to 826 m^2^/g (Fig. 6c). With the high surface area and abundant O/N sits (Supplementary Fig. 22), all these hyperporous carbons exhibited good electrochemical performance with specific capacitances higher than 450 F/g at 1 mV/s (Fig. 6d). The specific capacitances of hyperporous carbon are positively related to their specific surface area because the charge storage mechanism of carbon supercapacitors is electrical double-layer capacitance (EDLC). From C-Ben to C-Phl, the specific capacitance at 1 mV/s increases from 461 to 610 F/g, mainly from the increase of surface area. Based on the results above, a series of hyperporous carbons with abundant O/N sites are obtained by the general NaNH_2_ activation of different HCPs.

### Experimental feedback to ANN model

**Fig. 7:**
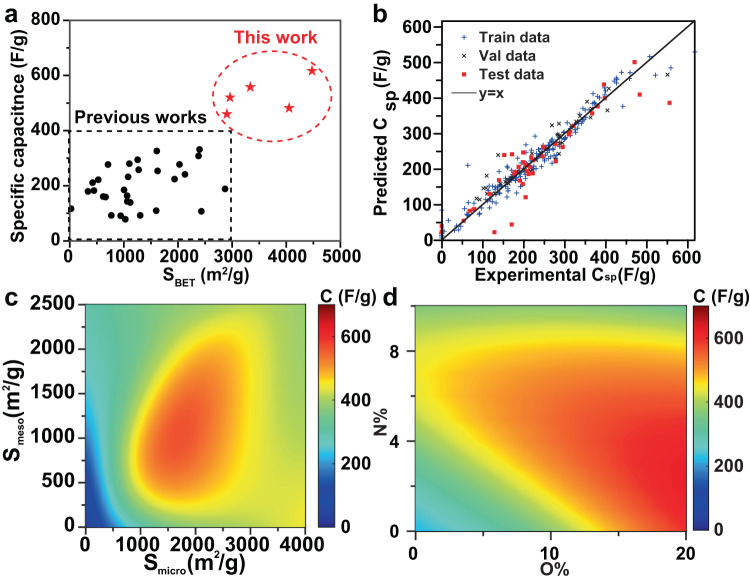
Experimental feedback to ANN model. **a** Comparison of the capacitances and BET surface areas reported in this work with previously reported values. For literature data, only those with high capacitances above 100 F/g were selected and plotted. **b** Correlated capacitance vs experimental capacitance for the ANN model with the additional experimental data. Train RMSE = 33.0, Val RMSE = 35.6, Test RMSE = 46.2. **c** Capacitance vs the surface areas of micropores and mesopores when doped with 2.3% nitrogen and 20% oxygen at 5 mV/s. **d** Specific capacitance vs O/N content for carbon materials with S_micro_ = 1710 m^2^/g, S_meso_ = 1050 m^2^/g at 5 mV/s.

Combining the ANN guideline with the experimental design, the hyperporous carbons reported here exhibited much higher specific capacitance values and BET surface areas than previously reported porous carbons for supercapacitors (Fig. 7a). The 5-fold learning curves of the ANN model with or without the experimental data were shown in Supplementary Fig. 23 and 24, respectively. The ANN based on the data points from the literature match with the present experiments but not fully (Supplementary Fig. 25) because hyperporous carbons surpass the boundaries of reported data plots. With additional experimental data collected in this work where structural and chemical features are similar to the best N/O co-doped carbon electrode predicted by ANN, we further reinforced the training of the machine-learning model. Thus, we reinforced the ML model with additional experimental data collected in this work (Supplementary Fig. 26). Especially the hyperporous carbons synthesized in this work surpass the surface area and specific capacitance boundaries of reported data plots, which can make up for the lack of hyperporous carbon supercapacitors in the database. As shown in Fig. 7b, with the addition of extra experimental data, ANN can still correlate the electrochemical performance over a wide range of specific capacitance (Train RMSE = 33.0, Val RMSE = 35.6, Test RMSE = 46.2.). The feedback from additional experimental data also helps ANN better predict the specific capacitance at a large surface area. According to the updated ANN prediction, the maximum capacitance can be achieved with the micropore surface area of 1710 m^2^/g, mesopore surface area of 1050 m^2^/g, 2.3 at% nitrogen-doping, and 20 at% oxygen-doping. Specific capacitance vs. O/N content for carbon materials with S_micro_ = 1710 m^2^/g, S_meso_ = 1050 m^2^/g at 5 mV/s is shown in Fig. 7d, which reveals a clear increase of capacitance with O content. 20 at% was set as a boundary of O content because no higher value has been reported for carbon supercapacitors. And with the increase of O content higher than 20 at%, the decrease in electronic conductivity needs to be considered, which will limit the rate performance of carbon supercapacitors. The capacitance versus micropore surface area and mesopore surface area at 5 mV/s (Fig. 7c) is predicted when a porous carbon electrode is doped with 2.3 at% nitrogen and 20 at% oxygen. Although the total surface area of N/O co-doped carbon electrode with the highest specific capacitance is smaller than experimental synthesis, the large ratio of mesopore surface area would enhance electrical conductivity and wettability of carbon electrodes to achieve a higher specific capacitance. Based on the reported porous carbons, the micropore surface area of 1710 m^2^/g with 2.3 at% nitrogen-doping and 20 at% oxygen-doping can be achieved by optimized carbonization-activation processes, while a mesopore surface area of 1050 m^2^/g is still challenging but worth to pursue a higher specific capacitance record of porous carbon-based supercapacitors.

## Discussion

Based on the ANN predicted specific capacitance boundary of N/O co-doped porous carbon supercapacitor, a series of hyperporous carbons featuring high surface areas up to 4476 m^2^/g and high O contents above 10 wt% were designed and synthesized via the sodium amide activation of HCPs at a medium temperature of 600 °C. The medium temperature activation is conducive to achieving a high O doping content. The hydroxyl groups in monomers are conducive to a higher surface area in the resulting hyperporous carbon products. The specific capacitance of C-Phl is as high as 610 F/g at a scan rate of 1 mV/s and 628 F/g at a specific current of 0.2 A/g in 1 M H_2_SO_4_ solution. C-Phl and C-Phl-700 having comparable pore structure and heteroatom content were used as model materials for the investigation of charge storage mechanisms. Based on the SPECS analysis, the high specific capacitance of C-Phl is mainly contributed by mesopores and diffusion processes. According to QENS results, the functional groups in C-Phl are more efficient in attracting the electrolyte components into the pores, which may be a decisive factor for the higher specific capacitance from mesopores and diffusion processes, while the faster dynamics of the electrolytes in C-Phl-700 correspond to the better rate performance of C-Phl-700. Carbon materials with higher mesopore surface area and higher O contents can be designed to expand the current limit in specific energy and rate performances of carbon-based supercapacitor devices. This work provides a machine learning assisted discovery of O-rich hyperporous carbon materials for supercapacitor application and a promising SPECS-QENS method for the charge storage mechanism investigation.

## Methods

### Materials

Anhydrous ferric chloride (≥98.0%), phloroglucinol (≥99.0%), dimethoxymethane (FDA, 99%), 1,2-dichloroethane (99.8%), benzene (99.8%), phenol (99.0–100.5%), resorcinol (≥99.0%), NaNH_2_ (98%) were purchased from Millipore Sigma. Carbon black (acetylene black) was purchased from Soltex. Nafion solution (5 wt% in isopropanol) was purchased from Ion Power. HCl (37%, aqueous solution), H_2_SO_4_ (98%), and isopropanol (99.9%) were purchased from Fisher Scientific. Hg/Hg_2_SO_4_ electrode (CHI 151) was purchased from CH Instruments accessories. Pt electrode (1 × 1 cm^2^) and glassy carbon electrode (5 mm in diameter, L type) were purchased from eBay.

### Preparation of HCP-Phl

6.50 g of anhydrous ferric chloride was added to a solution of phloroglucinol (2.00 g) and FDA (3.04 g) in 20 mL of 1,2-dichloroethane under stirring. The mixture was heated to 45 °C for 5 h and then to 80 °C for 19 h. The product was collected and washed with methanol by vacuum filtration and then washed with refluxed methanol in a Soxhlet extractor for 24 h. Then the product was dried at 60 °C in a vacuum oven.

### Preparation of HCP-Ben

9.75 g of anhydrous ferric chloride was added to a solution of benzene (1.56 g) and FDA (4.56 g) in 20 mL of 1,2-dichloroethane under stirring. The mixture was heated to 45 °C for 5 h and then to 80 °C for 19 h. The product was collected and washed with methanol by vacuum filtration and then washed with refluxed methanol in a Soxhlet extractor for 24 h. Then the product was dried at 60 °C in a vacuum oven.

### Preparation of HCP-Phe

6.50 g of anhydrous ferric chloride was added to a solution of phenol (1.98 g) and FDA (3.04 g) in 20 mL of 1,2-dichloroethane under stirring. The mixture was heated to 45 °C for 5 h and then to 80 °C for 19 h. The product was collected and washed with methanol by vacuum filtration and then washed with refluxed methanol in a Soxhlet extractor for 24 h. Then the product was dried at 60 °C in a vacuum oven.

### Preparation of HCP-Res

6.50 g of anhydrous ferric chloride was added to a solution of resorcinol (2.00 g) and FDA (3.04 g) in 20 mL of 1,2-dichloroethane under stirring. The mixture was heated to 45 °C for 5 h and then to 80 °C for 19 h. The product was collected and washed with methanol by vacuum filtration and then washed with refluxed methanol in a Soxhlet extractor for 24 h. Then the product was dried at 60 °C in a vacuum oven.

### Preparation of hyperporous carbons

Taking the synthesis of C-Phl as an example, 0.50 g of HCP was mixed with 1.00 g of NaNH_2_ by hand grinding in an argon glove box (NaNH_2_ is reactive to water or oxygen, that is why NaNH_2_ and hypercrosslinked polymer must be mixed in the 99.999% argon-filled glove box with an oxygen concentration below 1 ppm and water concentration below 1 ppm before activation). The mixture was heated to 350 °C for 1 h with a heating rate of 5 °C/min and then to 600 °C for 2 h with a heating rate of 5 °C/min in a tube furnace (N_2_ atmosphere). After cooling to 20 °C, the carbon product was washed with water and collected by vacuum filtration. Then the product was soaked in 3 M HCl solution, heated to 60 °C for 5 h, and washed with water again by vacuum filtration. Finally, the product was dried at 100 °C in a vacuum oven to obtain the C-Phl. Other hyperporous carbons were synthesized using their corresponding HCPs under the same condition.

C-Phl-700 and C-Phl-500 were synthesized by changing the heating temperature from 600 °C to 700 °C and 500 °C, respectively. All the other treatments were the same as C-Phl.

### Electrode preparation and electrochemical protocol

14 mg of hyperporous carbon, 4 mg of carbon black (acetylene black), 40 μl of Nafion solution (5 wt%), and 0.96 ml of isopropanol were mixed to obtain an ink. 10 μl of the ink was drop cast onto a polished glassy carbon rod as the working electrode. The diameter of the glassy carbon rod is 5 mm, which provides an electrode area of 0.2 cm^2^. The mass ratio of the active material, conductive carbon, and binder in the working electrode is 7:2:1. The mass loading of hyperporous carbon on the working electrode was about 0.7 mg cm^−2^. For a high loading of 4.2 mg cm^−2^, 60 μl of the ink was drop-cast onto a polished glassy carbon rod as the working electrode. The thickness of the electrodes (without current collectors) at a low mass loading of 0.7 mg/cm^2^ is 30 μm. For a high mass loading of 4.2 mg/cm^2^, the thickness is 157 μm. Hg/Hg_2_SO_4_ electrode (0.64 V vs. NHE) filled with a concentrated K_2_SO_4_ supporting solution was used as the reference electrode, 1 M H_2_SO_4_ aqueous solution (~60 mL) as an electrolyte, and a piece of platinum mesh as the counter electrode (Supplementary Fig. 26). The electrochemical data were collected on a CHI 760E instrument at 20 °C. No climatic/environmental chamber was used. At least three cells were tested for a single electrochemical experiment to ensure good reproducibility. The working electrode was initially aged by cyclic voltammetry for 200 cycles at 100 mV/s, between −0.6 and 0.4 V vs. Hg/Hg_2_SO_4,_ to establish a stable cycling behavior. Then, cyclic voltammetry was performed at a series of scan rates from 1 to 500 mV/s between −0.6 and 0.4 V vs. Hg/Hg_2_SO_4_. The average capacitances were calculated from their CV data according to Eq. (7):

7 $$C=\frac{\int {i}_{m}{EdE}}{2v\Delta E}$$

where C (F/g) is the specific capacitance, i_m_ (A/g) and E (V) are specific current and potential in voltammogram, v (V/s) is the scan rate, and ΔE (V) is the potential window. The mass considered for calculating the specific currents (A/g) and capacitance (F/g) values refer to the active material’s mass.

GCD tests were performed at a series of specific currents from 0.2 to 100 A/g between −0.6 and 0.4 V vs. Hg/Hg_2_SO_4_. SPECS measurements were performed using a potential step of 25 mV between −0.6 and 0.4 V with an equilibration time of 300 s. Voltage hold test using three-electrode cells at 20 °C by applying a constant up-limit potential (0.4 V vs Hg/Hg_2_SO_4_) during aging for up to 500 h. Every 10 h, four GCD cycles were performed between −0.6 V and 0.4 V vs Hg/Hg_2_SO_4_ using a specific current of 20 A/g. The AC impedance measurements were carried out in three-electrode cells at open circuit potential with an amplitude of 5 mV. The frequency range of 0.01 to 100,000 Hz was used. The number of data points per decade of frequency was 12. An open-circuit voltage time of 2 s was applied before carrying out the EIS measurement.

### Characterization techniques

The X-ray diffraction (XRD) patterns were collected on a PANalytical Empyrean diffractometer with Cu Ka radiation, operated at 45 kV and 40 mA. X-ray photoelectron spectroscopy (XPS) was performed on a PHI 3056 spectrometer equipped with an Al anode source operated at 15 kV, an applied power of 350 W, and a pass energy of 93.5 eV. Raman spectroscopy was performed on a Renishaw inVia confocal Raman microscope with a 532 nm laser. N_2_ adsorption-desorption experiments were performed on a 3 Flex Micromeritics Setup at 77 K. ^13^C{^1^H} cross-polarization magic-angle spinning (CPMAS) experiment on a Bruker Biospin AVANCE I spectrometer, operating at 9.4 T equipped with a 4 mm double-resonance MAS probe. A MAS rate of 7.5 kHz was used. STEM imaging was performed on a probe-corrected JOEL NEOARM electron microscope at 80 kV.

### Description of the QENS samples and experimental details

The highly porous carbons defined in the main manuscript as C-Phl and C-Phl-700 have been investigated for these experiments. The carbons were soaked overnight in 1 M H_2_SO_4_ solution and separated by fast vacuum filtration to remove free and loosely bound water. The final samples were defined as wet samples and had molar fractions of carbon of around 20% and 13% in C-Phl and C-Phl-700 respectively. Samples of dry carbon, that is, not soaked in the electrolyte, were also prepared, and dried at 140 °C under vacuum for 72 h.

The QENS experiments were performed at the BASIS spectrometer, which provides an energy resolution of 3.7 μeV (full-width at half-maximum for the *Q*-averaged resolution value) and an accessible dynamical range of ±100 μeV. For the experiments, ~40 mg of the wet samples were placed in Au-coated Al flat plates, and sealed with indium gaskets. For the dry samples, ~200 mg of each material was placed in Al flat plates and sealed with indium gaskets. All these procedures were conducted in an Ar-filled glovebox.

The samples were initially cooled down to 20 K, and full QENS spectra were collected to be used as the instrumental resolutions. Then, temperature-dependent elastic scattering intensity scans were collected between 20 K and 300 K with 1 K steps, and full QENS spectra were collected at 300 K. For background subtraction, data from empty sample holders (both Au-coated and not Au-coated) were collected at 300 K.

## Supplementary information

Supplementary information

## Data Availability

The data that support the findings of this study have been included in the main text and Supplementary Information. Other relevant original data is available from the corresponding author upon reasonable request. Source data are provided with this paper.

## Source data

Source Data

## Code availability

The code for ANN training is provided in the Supplementary Source Data file.
