# Supplementary material for: Machine-learning-assisted material discovery of oxygen-rich highly porous carbon active materials for aqueous supercapacitors
Source: Nat Commun. 2023 Aug 1;14:4607. doi: 10.1038/s41467-023-40282-1 (PMC10393944; doi:10.1038/s41467-023-40282-1)
Supplement: Supplementary file 1 — Supplementary information [file 41467_2023_40282_MOESM1_ESM.pdf]

Machine-Learning-Assisted Material Discovery of Oxygen-Rich Highly Porous Carbon Active Materials for Aqueous Supercapacitors

Tao Wang,[1,2] Runtong Pan,[3] Murillo L. Martins,[4] Jinlei Cui,[5] Zhennan Huang,[6] Bishnu P. Thapaliya,[1,2] Chi-Linh Do-Thanh,[2] Musen Zhou,[3] Juntian Fan,[2] Zhenzhen Yang,[1,2] Miaofang Chi,[6] Takeshi Kobayashi,[5] Jianzhong Wu,[3] Eugene Mamontov,[4] Sheng Dai[1,2]*

[1] *Chemical Sciences Division, Oak Ridge National Laboratory, Oak Ridge, TN 37831, USA*

[2] *Department of Chemistry, Institute for Advanced Materials and Manufacturing, University of Tennessee, Knoxville, Tennessee 37996, USA*

[3] *Department of Chemical and Environmental Engineering, University of California, Riverside, Riverside, California 92521, USA*

[4] *Neutron Scattering Division, Oak Ridge National Laboratory, Oak Ridge, Tennessee 37831, USA*

[5] *U.S. DOE Ames Laboratory, Ames, Iowa 50011, USA*

[6] *Center for Nanophase Materials Sciences, Oak Ridge National Laboratory. Oak Ridge, Tennessee 37831, USA*

[*] *Email: dais@ornl.gov*

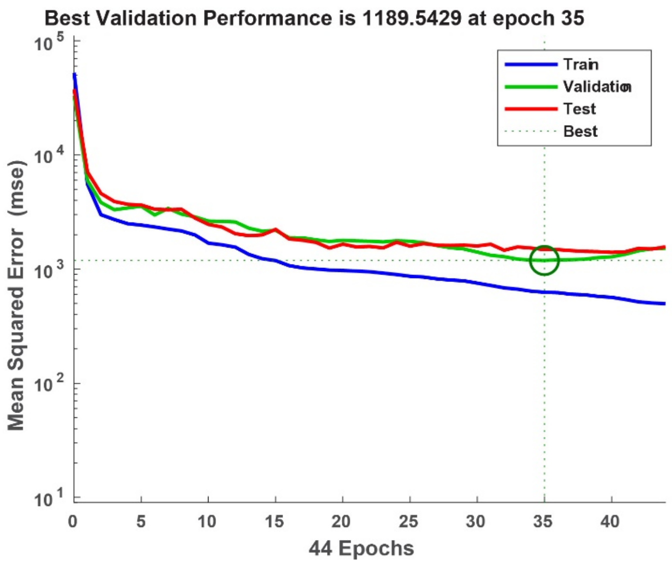

Supplementary Figure 1| The learning curves of the ANN model without the experimental data.

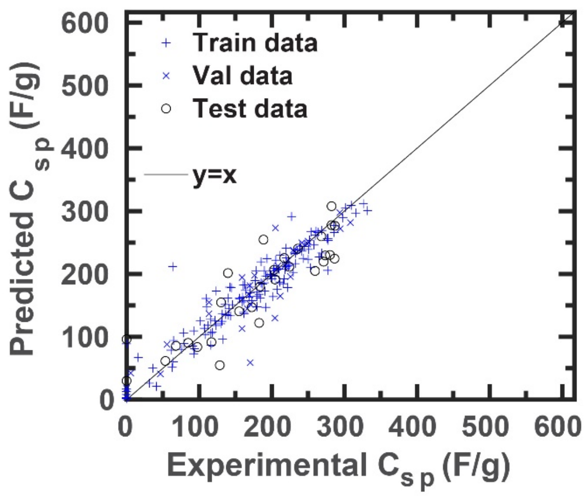

Supplementary Figure 2| Correlated capacitance vs experimental capacitance for the ANN model based on the data plots from the literature. Train RMSE=25.0, Val RMSE=34.5, Test RMSE=38.5. C_{sp} means "specific capacitance".

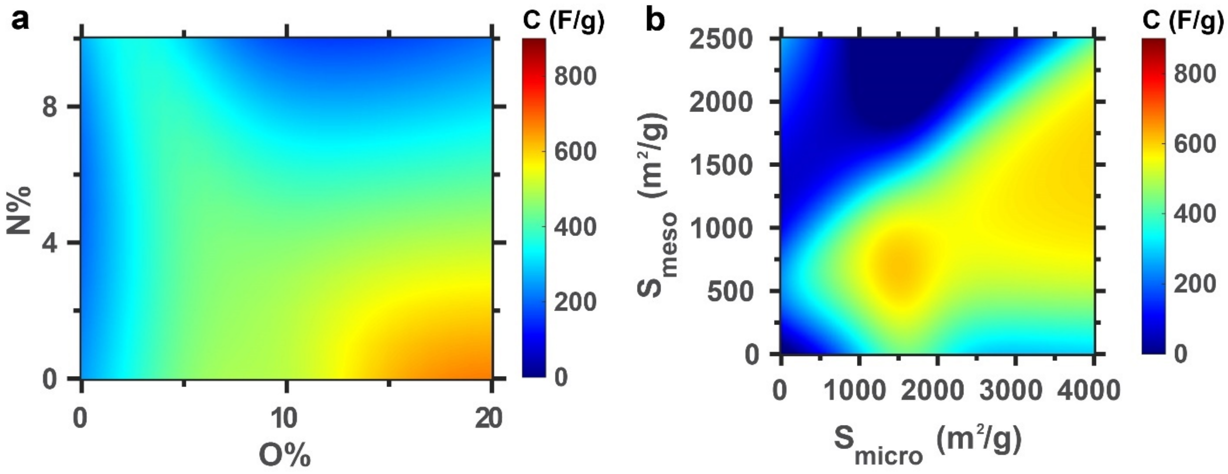

Supplementary Figure 3| a, Specific capacitance vs O/N content for carbon materials with S_{micro}=1502 m^2/g, S_{meso}=687 m^2/g at 5 mV/s. b, Capacitance vs the surface areas of micropores and mesopores when doped with 0.5% nitrogen and 20% oxygen at 5 mV/s.

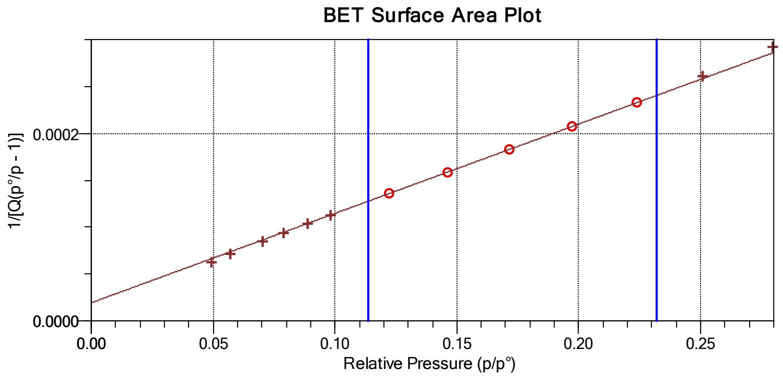

Supplementary Figure 4| BET surface area plots calculated by the BET equation.

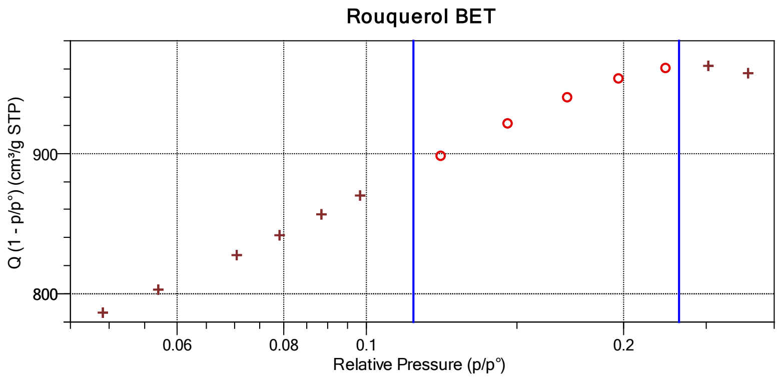

Supplementary Figure 5| Rouquerol plots were used to select a reasonable pressure range for BET surface area calculation where the term Q(1 – p/p°) continuously increases.

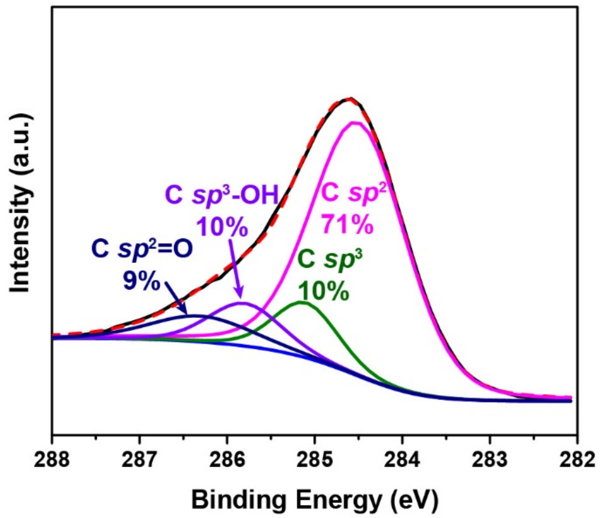

Supplementary Figure 6| C 1*s* XPS spectra of C-Phl.

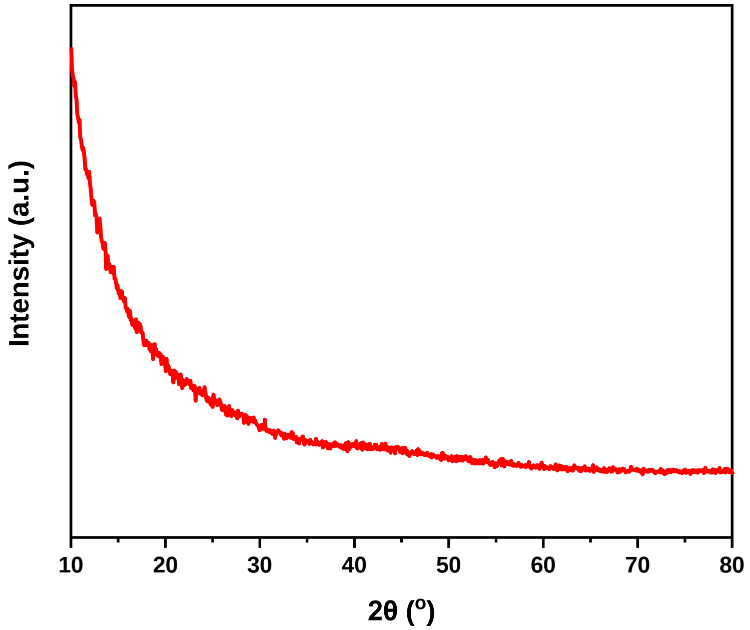

Supplementary Figure 7| Powder XRD pattern of C-Phl.

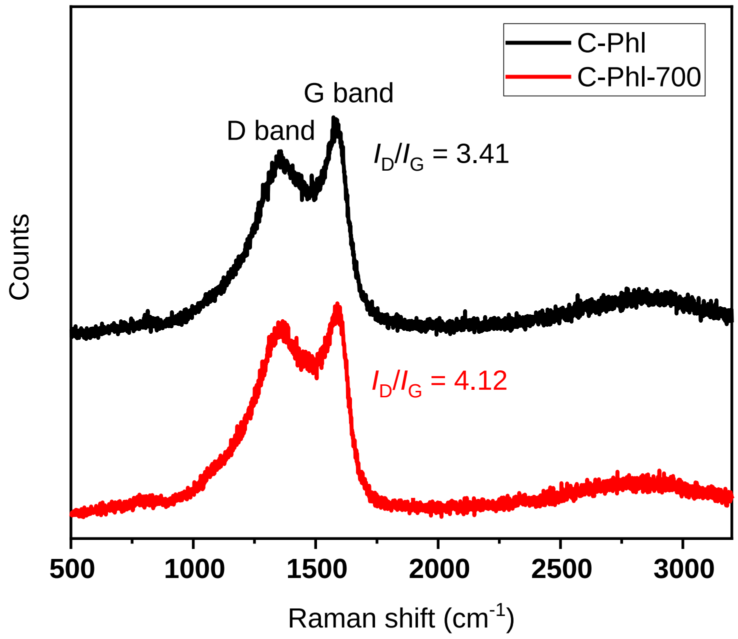

Supplementary Figure 8| Raman spectra of C-Phl and C-Phl-700. Raman spectra reveal the signals from the defects in the graphitic lattice (D band) at 1353 cm^{-1}, and the in-plane vibration of the sp^2 carbon network (G band) at 1590 cm^{-1}. The high area ratio of D band to G band (I_D/I_G) above 3 indicates the amorphous nature of these carbon products.

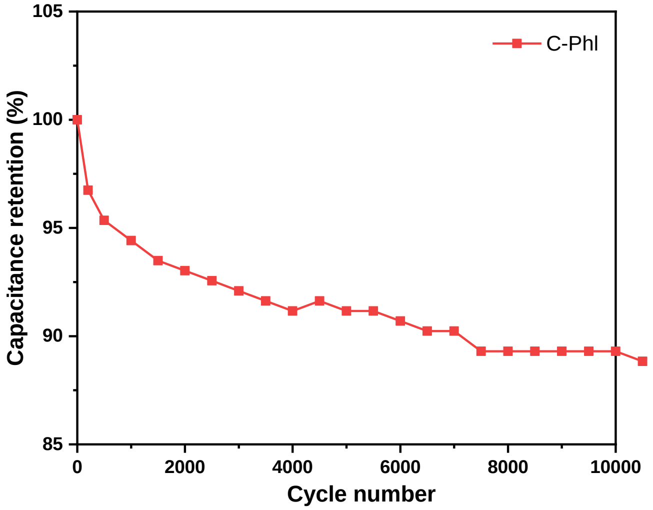

Supplementary Figure 9| Cycling performance of C-Phl at 20 A/g using three-electrode cells at 20 °C. The mass loading of the working electrode is 0.7 mg/cm^2.

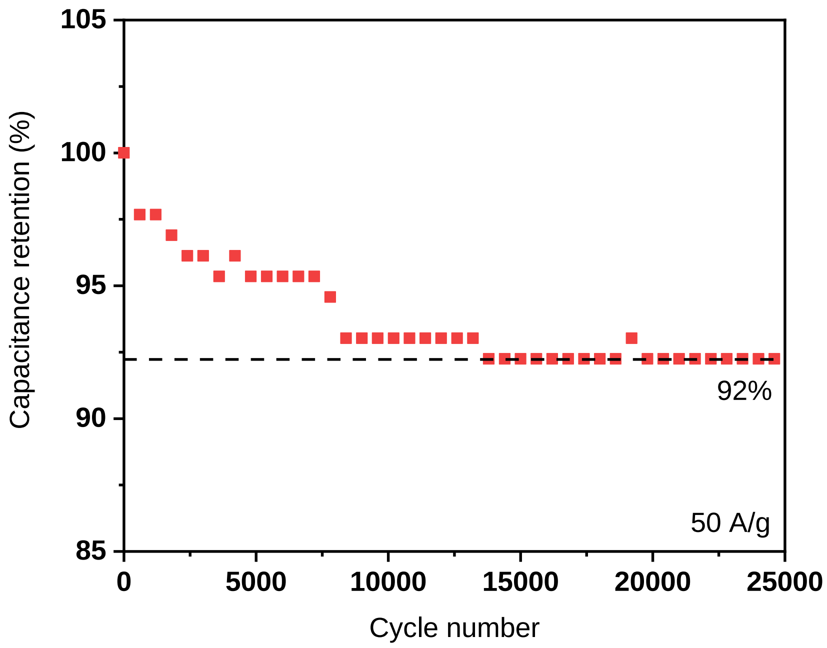

Supplementary Figure 10| GCD Cycling performance of C-Phl at 50 A/g using three-electrode cells at 20 °C. The mass loading of the working electrode is 0.7 mg/cm^2.

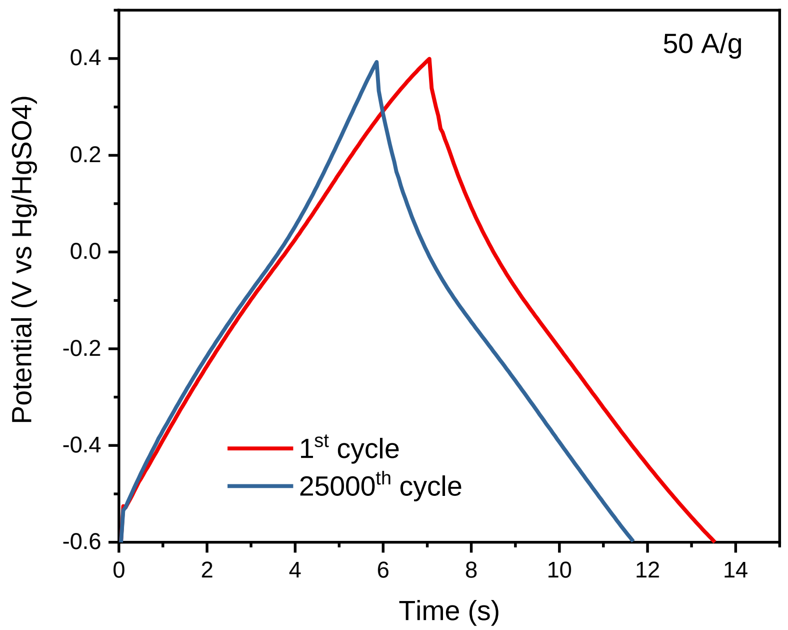

Supplementary Figure 11. 1st and 25000th GCD curves of C-Phl at 50 A/g using three-electrode cells at 20 °C. The mass loading of the working electrode is 0.7 mg/cm^2.

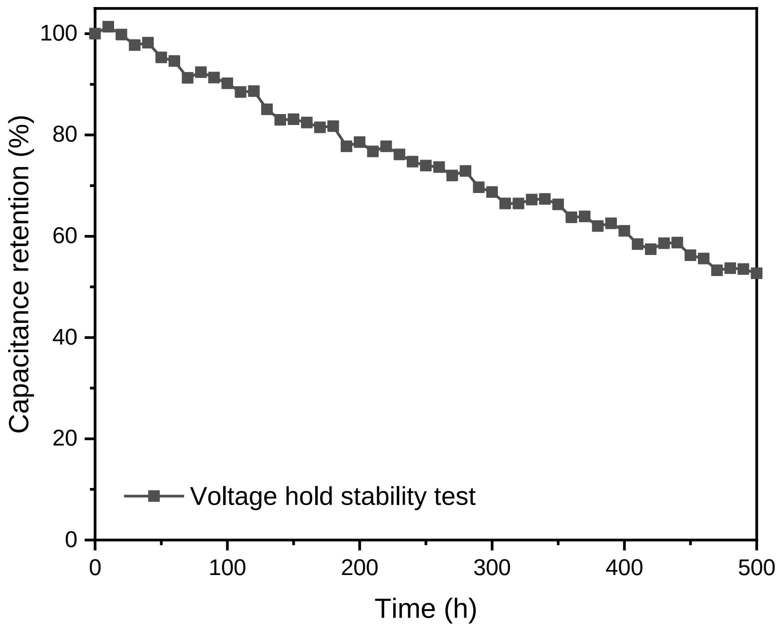

Supplementary Figure 12| Voltage hold test using three-electrode cells at 20 °C by applying a constant up-limit potential (0.4 V vs Hg/Hg_2SO_4) during aging for up to 500 h. Every 10 h, four GCD cycles were performed between -0.6 V and 0.4 V vs Hg/Hg_2SO_4 using a specific current of 20 A/g. The mass loading of the working electrode is 0.7 mg/cm^2.

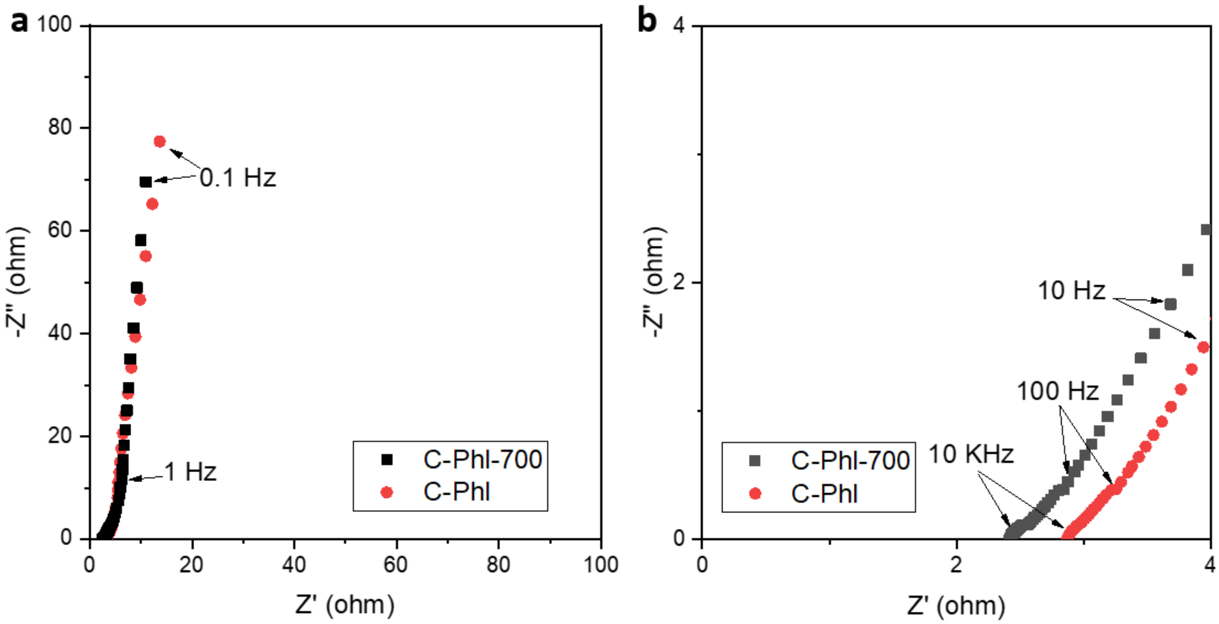

Supplementary Figure 13| a) Nyquist plots of three-electrode cells with C-Phl and C-Phl-700 as active materials at 20 °C. The mass loading of the working electrode is 0.7 mg/cm^2. b) Zoom-in Nyquist plots at the high-frequency region of impedance.

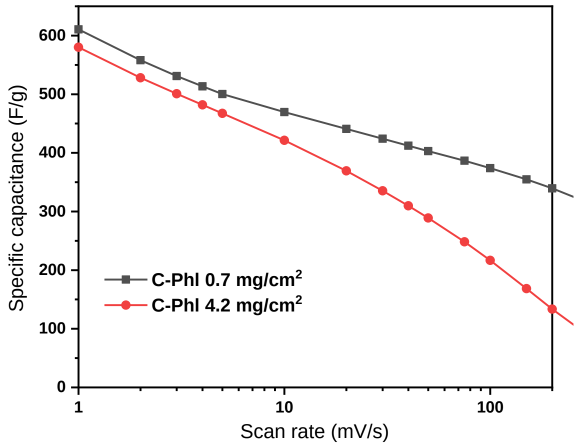

Supplementary Figure 14| Specific capacitance of C-Phl at different mass loadings. Three-electrode cells were used and tested at 20 °C.

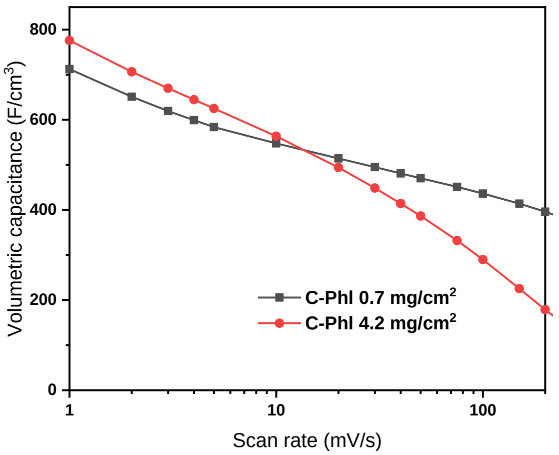

Supplementary Figure 15| Volumetric capacitance of C-Phl at different mass loadings. Three-electrode cells were used and tested at 20 °C.

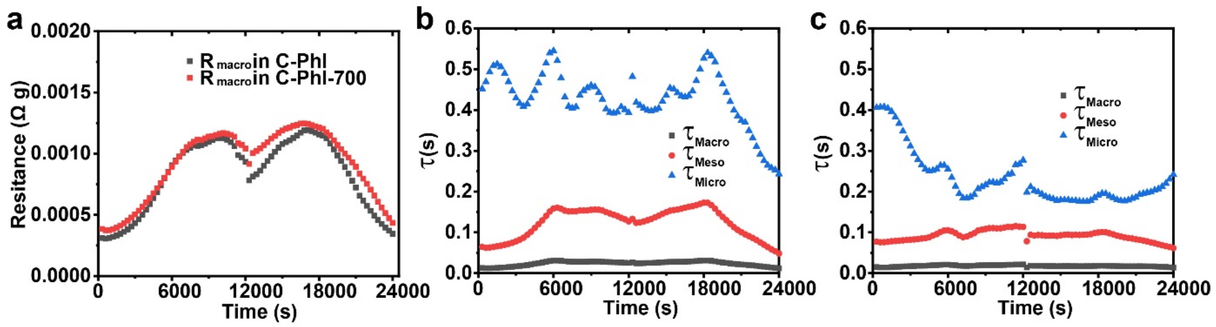

Supplementary Figure 16| Parameters fitted from the SPECS data of C-Phl and C-Phl-700.

a) Macropore resistances; b) time constants of C-Phl; (c) time constants of C-Phl-700. The discontinuities in the curves in Figure 4c, Supplementary Figure 16 b and c are due to the change of current direction at 12000s as shown in Figure 4a.

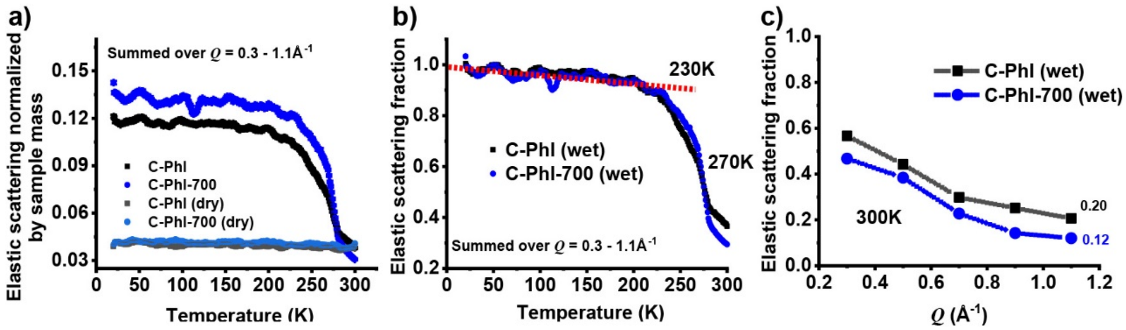

Supplementary Figure 17| Elastic scattering intensity scans (a) normalized by the samples' mass for the dry and wet samples, and (b) for the wet samples with the datapoints at T = 20 K normalized to unity. In both cases, the data are summed over $Q = 0.3–1.1Å^{-1}$. In (c), the Q-dependences of the fraction of the elastic scattering intensities in the QENS signals at 300 K are presented. The numbers on the right-hand side indicate the elastic scattering fraction at $Q = 1.1$ $Å^{-1}$.

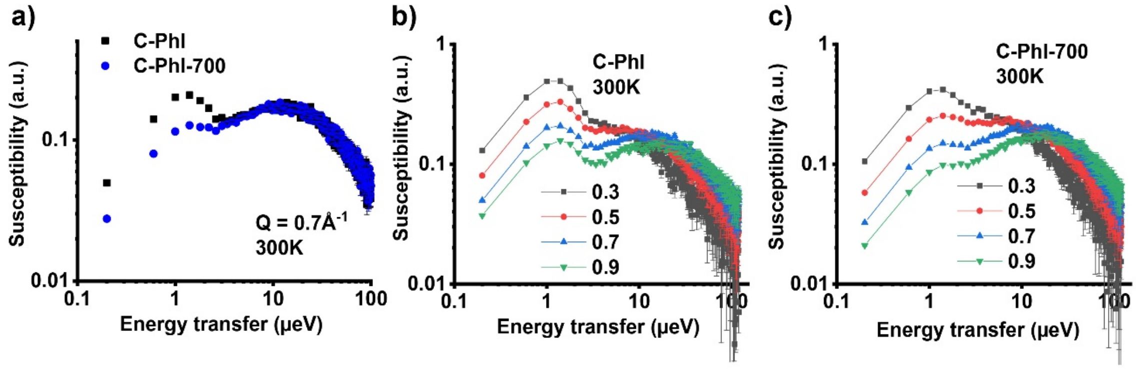

Supplementary Figure 18| QENS spectra collected at 300K presented as (a) the dynamic susceptibilities. The data at $Q = 0.7$ $Å^{-1}$ are shown as representative examples of the behavior of the sample. In (b) and (c), the dynamic susceptibilities are presented at different Q-values.

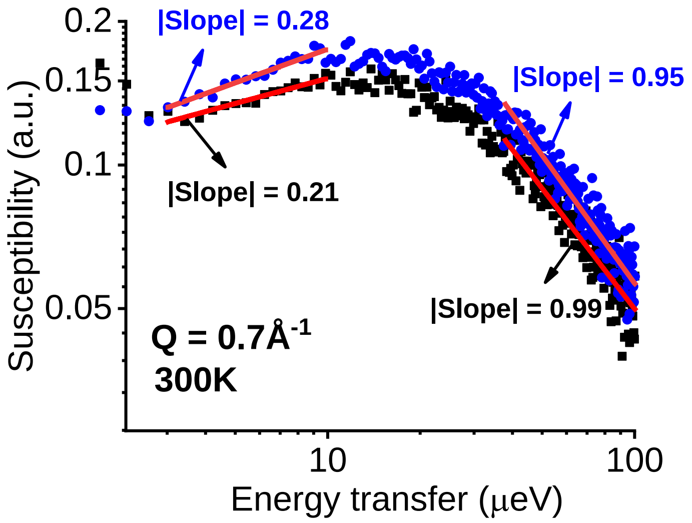

Supplementary Figure 19| The relaxations above 10 μeV previously shown in Supplementary Figure 18a are reproduced with the slopes of the low and high-frequency sides being depicted.

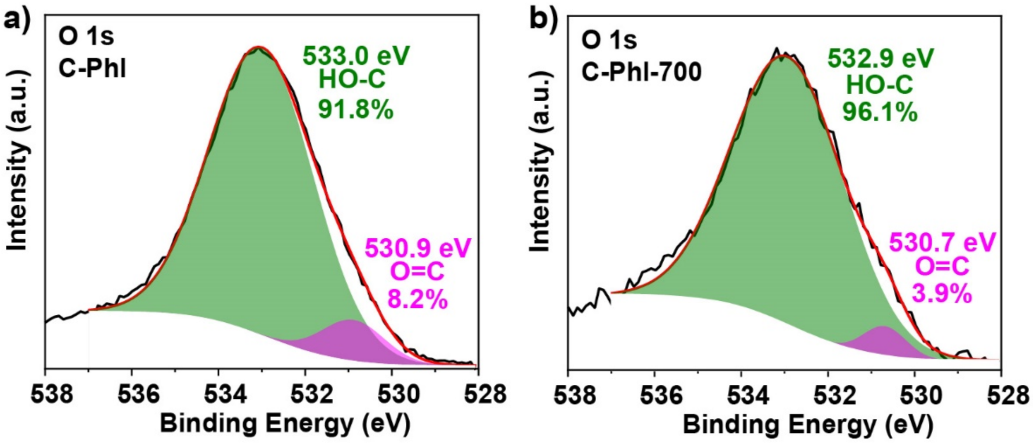

Supplementary Figure 20| The analysis of O 1s peaks for C-Phl (a) and C-Phl-700 (b).

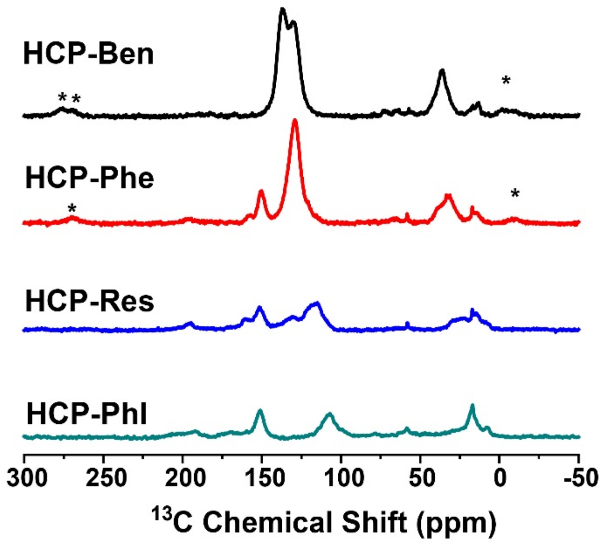

Supplementary Figure 21| ^{13}C CP/MAS NMR of HCPs.

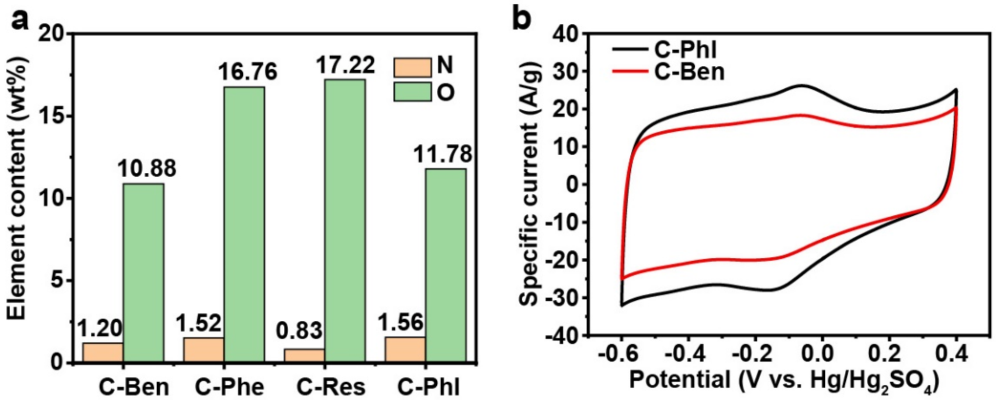

Supplementary Figure 22| N and O contents in hyperporous carbons (a) and CV curves of C-Phl and C-Ben at 50 mV/s (b). Three-electrode cells were used and tested at 20 °C. The mass loading of the working electrode is 0.7 mg/cm^2.

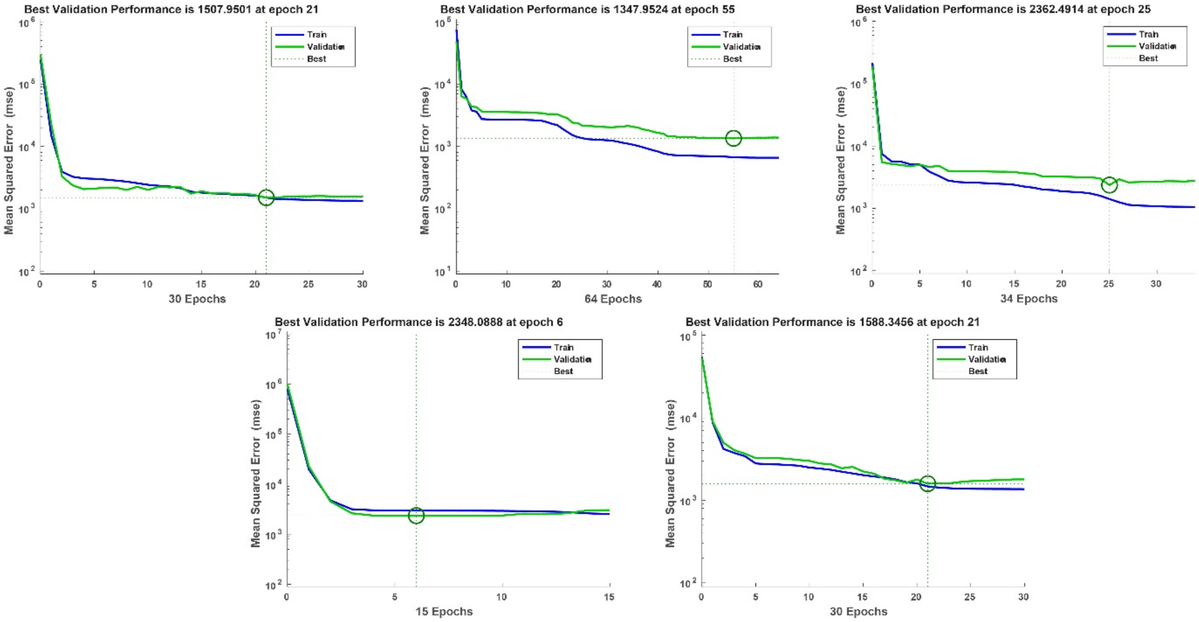

Supplementary Figure 23| The 5-fold learning curves of the ANN model without the experimental data. The best validation MSEs range from 1348 to 2362, corresponding to RMSEs from 36.7 to 48.6.

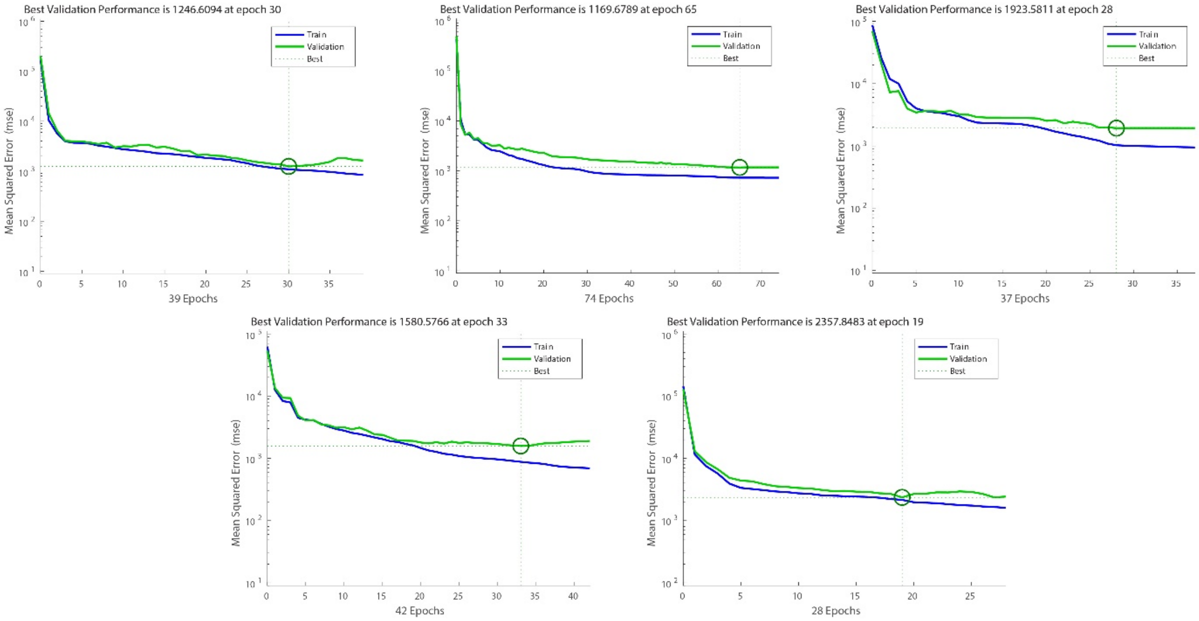

Supplementary Figure 24| The 5-fold learning curves of the ANN model with the experimental data. The best validation MSEs range from 1170 to 2357, corresponding to RMSEs from 36.2 to 48.5.

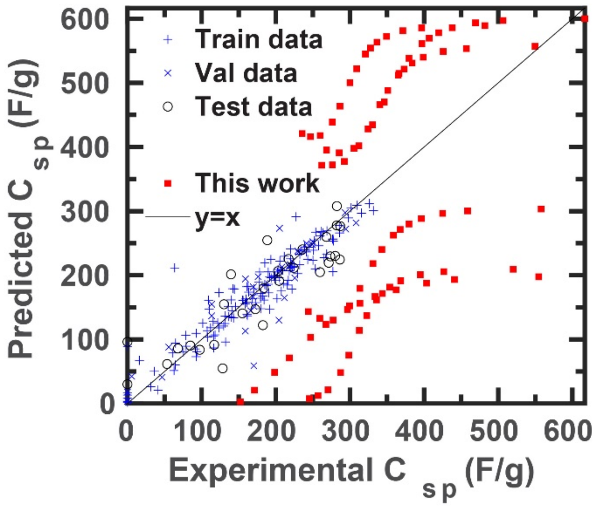

Supplementary Figure 25| Correlated capacitance vs experimental capacitance for the ANN model without the experimental data. Train RMSE=25.0, Val RMSE=34.5, Test RMSE=38.5, Experimental data (This work) RMSE=165.8. C_{sp} means “specific capacitance”.

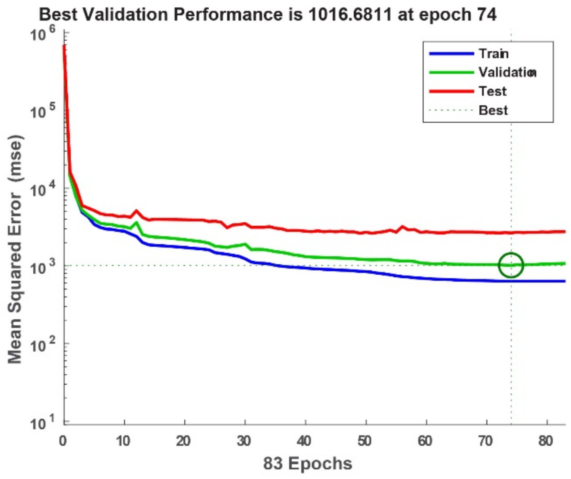

Supplementary Figure 26| The learning curves of the ANN model with additional experimental data from this work.

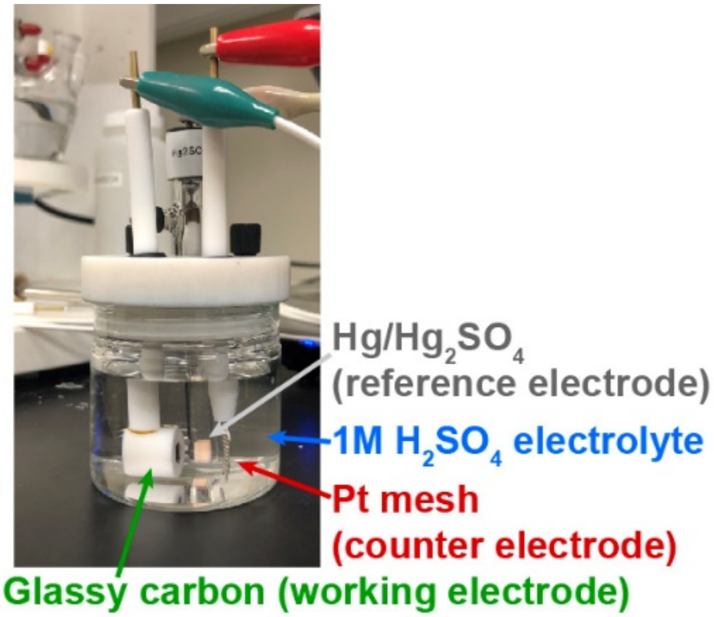

Supplementary Figure 26| Photographic picture of the cell for electrochemical tests.

Supplementary Table 1. Input and output data used in different machine learning models

#	C_{sp} (F/g)	electrolyte	υ (mV/s)	SA_{micro} (m^2/g)	SA_{meso} (m^2/g)	O (at%)	N (at%)
1	0	6M KOH	1	0	0	0	0
2	0	6M KOH	300	0	0	0	0
3	0	6M KOH	500	0	0	0	0
4	0	6M KOH	1	0	0	17	15.6
5	0	6M KOH	300	0	0	17	15.6
6	0	6M KOH	500	0	0	17	15.6
7	0	6M KOH	1	0	0	8.5	7.8
8	0	6M KOH	300	0	0	8.5	7.8
9	0	6M KOH	500	0	0	8.5	7.8
10	179.60	6M KOH	1	120	216	0	0
11	172.40	6M KOH	2	120	216	0	0
12	166.30	6M KOH	5	120	216	0	0

13	155.00	6M KOH	10	120	216	0	0
14	211.60	6M KOH	1	107	315	0	0
15	201.60	6M KOH	2	107	315	0	0
16	184.20	6M KOH	5	107	315	0	0
17	172.60	6M KOH	10	107	315	0	0
18	277.00	6M KOH	1	153	553	0	0
19	259.60	6M KOH	2	153	553	0	0
20	229.50	6M KOH	5	153	553	0	0
21	198.10	6M KOH	10	153	553	0	0
22	280.10	6M KOH	1	200	900	0	0
23	273.50	6M KOH	2	200	900	0	0
24	265.20	6M KOH	5	200	900	0	0
25	250.10	6M KOH	10	200	900	0	0
26	224.15	6M KOH	2	735	1200	0	0
27	202.99	6M KOH	5	735	1200	0	0
28	189.89	6M KOH	10	735	1200	0	0
29	176.24	6M KOH	20	735	1200	0	0
30	144.14	6M KOH	50	735	1200	0	0
31	113.60	6M KOH	100	735	1200	0	0
32	143.09	6M KOH	10	417	645	6.14	8.28
33	118.00	6M KOH	50	417	645	6.14	8.28
34	97.41	6M KOH	100	417	645	6.14	8.28
35	36.15	6M KOH	400	417	645	6.14	8.28
36	163.62	6M KOH	10	219	840	5.83	5.2
37	141.39	6M KOH	50	219	840	5.83	5.2
38	123.27	6M KOH	100	219	840	5.83	5.2
39	62.76	6M KOH	400	219	840	5.83	5.2
40	139.59	6M KOH	10	173	944	5.23	3.69
41	123.52	6M KOH	50	173	944	5.23	3.69
42	110.26	6M KOH	100	173	944	5.23	3.69

43	64.69	6M KOH	400	173	944	5.23	3.69
44	276.38	6M KOH	5	1283	764	8.43	0.69
45	249.80	6M KOH	10	1283	764	8.43	0.69
46	239.01	6M KOH	20	1283	764	8.43	0.69
47	222.43	6M KOH	50	1283	764	8.43	0.69
48	204.46	6M KOH	100	1283	764	8.43	0.69
49	190.26	6M KOH	150	1283	764	8.43	0.69
50	179.47	6M KOH	200	1283	764	8.43	0.69
51	168.44	6M KOH	250	1283	764	8.43	0.69
52	286.01	6M KOH	5	1574	187	12.28	2.96
53	268.15	6M KOH	10	1574	187	12.28	2.96
54	249.17	6M KOH	20	1574	187	12.28	2.96
55	208.33	6M KOH	50	1574	187	12.28	2.96
56	159.75	6M KOH	100	1574	187	12.28	2.96
57	131.32	6M KOH	150	1574	187	12.28	2.96
58	282.26	6M KOH	5	1556	205	11.72	2.45
59	282.35	6M KOH	5	1320	621	10.52	1.42
60	205.06	6M KOH	5	1072	661	8.39	0.65
61	188.58	6M KOH	5	1990	879	0	0
62	232.27	6M KOH	5	636	442	0	0
63	222.77	6M KOH	10	636	442	0	0
64	202.29	6M KOH	20	636	442	0	0
65	185.15	6M KOH	50	636	442	0	0
66	155.41	6M KOH	100	636	442	0	0
67	185.11	6M KOH	5	713	290	0	0
68	294.33	6M KOH	5	971	282	7.87	1.99
69	277.32	6M KOH	5	633	1394	5.4	0.36
70	331.72	6M KOH	5	1227	1170	7.87	2.72
71	315.50	6M KOH	10	1227	1170	7.87	2.72
72	297.85	6M KOH	20	1227	1170	7.87	2.72

73	260.99	6M KOH	50	1227	1170	7.87	2.72
74	219.73	6M KOH	100	1227	1170	7.87	2.72
75	205.49	6M KOH	200	1227	1170	7.87	2.72
76	116.85	6M KOH	2	0	24	0	0
77	79.11	6M KOH	5	0	24	0	0
78	68.03	6M KOH	10	0	24	0	0
79	61.20	6M KOH	20	0	24	0	0
80	53.48	6M KOH	50	0	24	0	0
81	46.58	6M KOH	100	0	24	0	0
82	41.30	6M KOH	200	0	24	0	0
83	31.42	6M KOH	500	0	24	0	0
84	257.94	6M KOH	2	115	1158	0	0
85	244.31	6M KOH	5	115	1158	0	0
86	238.34	6M KOH	10	115	1158	0	0
87	232.37	6M KOH	20	115	1158	0	0
88	224.65	6M KOH	50	115	1158	0	0
89	216.90	6M KOH	100	115	1158	0	0
90	207.36	6M KOH	200	115	1158	0	0
91	187.26	6M KOH	500	115	1158	0	0
92	326.17	6M KOH	2	327	1280	6.8	4.8
93	309.45	6M KOH	5	327	1280	6.8	4.8
94	301.53	6M KOH	10	327	1280	6.8	4.8
95	293.59	6M KOH	20	327	1280	6.8	4.8
96	285.63	6M KOH	50	327	1280	6.8	4.8
97	277.62	6M KOH	100	327	1280	6.8	4.8
98	268.66	6M KOH	200	327	1280	6.8	4.8
99	250.54	6M KOH	500	327	1280	6.8	4.8
100	142.17	6M KOH	10	250	52	12.18	0
101	219.60	6M KOH	5	275	349	8.34	0
102	206.82	6M KOH	10	275	349	8.34	0

103	180.00	6M KOH	50	275	349	8.34	0
104	161.31	6M KOH	100	275	349	8.34	0
105	182.58	6M KOH	5	437	10	0	0
106	161.70	6M KOH	50	437	10	0	0
107	158.97	6M KOH	100	437	10	0	0
108	221.86	6M KOH	5	501	25	0	0
109	191.76	6M KOH	50	501	25	0	0
110	182.59	6M KOH	100	501	25	0	0
111	159.09	6M KOH	5	579	83	0	0
112	139.68	6M KOH	50	579	83	0	0
113	136.66	6M KOH	100	579	83	0	0
114	170.51	6M KOH	10	457	126	0	0
115	101.47	6M KOH	100	457	126	0	0
116	160.84	6M KOH	10	429	188	0	0
117	115.52	6M KOH	100	429	188	0	0
118	253.90	6M KOH	10	1118	504	6.22	0
119	203.05	6M KOH	100	1118	504	6.22	0
120	116.25	6M KOH	30	454	98	14.34	5.77
121	129.80	6M KOH	30	160	497	14.67	3.85
122	218.12	6M KOH	10	259	633	17.19	3.68
123	204.33	6M KOH	20	259	633	17.19	3.68
124	189.73	6M KOH	30	259	633	17.19	3.68
125	169.17	6M KOH	50	259	633	17.19	3.68
126	121.63	6M KOH	100	259	633	17.19	3.68
127	219.55	6M KOH	10	1052	253	7.81	4.36
128	286.41	6M KOH	5	1462	327	5.35	6.05
129	271.37	6M KOH	10	1462	327	5.35	6.05
130	253.23	6M KOH	20	1462	327	5.35	6.05
131	215.46	6M KOH	50	1462	327	5.35	6.05
132	170.36	6M KOH	100	1462	327	5.35	6.05

133	243.03	6M KOH	10	1133	279	7.97	4.6
134	218.08	6M KOH	10	897	233	7.69	3.68
135	211.06	6M KOH	10	567	293	7.54	2.91
136	204.29	6M KOH	10	824	298	7.2	8.46
137	209.60	6M KOH	10	1307	258	5.88	4.69
138	193.37	6M KOH	10	1099	182	5.95	2.86
139	132.27	6M KOH	10	1412	66	7.89	8.71
140	55.71	6M KOH	20	1412	66	7.89	8.71
141	16.13	6M KOH	50	1412	66	7.89	8.71
142	5.72	6M KOH	100	1412	66	7.89	8.71
143	91.28	6M KOH	10	889	51	10.91	8.61
144	78.59	6M KOH	10	952	74	8.38	7.33
145	92.56	6M KOH	10	1260	41	9.79	7.14
146	92.60	6M KOH	10	752	16	12.57	8.83
147	107.39	6M KOH	10	2316	115	4.94	5.1
148	109.51	6M KOH	10	1534	63	6.68	3.47
149	241.41	6M KOH	10	1809	319	12.45	7.88
150	308.22	6M KOH	5	1976	403	7.91	6.13
151	286.94	6M KOH	10	1976	403	7.91	6.13
152	263.59	6M KOH	20	1976	403	7.91	6.13
153	234.99	6M KOH	50	1976	403	7.91	6.13
154	199.04	6M KOH	100	1976	403	7.91	6.13
155	252.16	6M KOH	10	1552	369	8.37	5.8
156	170.33	6M KOH	10	562	128	11.09	9.23
157	227.24	6M KOH	10	779	1003	8.16	4.52
158	211.43	6M KOH	10	443	804	9.83	2.1
159	93.58	6M KOH	50	517	61	5.16	13.68
160	128.36	6M KOH	50	640	184	6.25	9.57
161	75.50	6M KOH	50	563	120	8.49	5.38
162	178.10	6M KOH	20	680	641	7.84	7.02

163	162.84	6M KOH	50	680	641	7.84	7.02
164	130.15	6M KOH	100	680	641	7.84	7.02
165	63.69	6M KOH	50	0	1082	0	0
166	268.95	6M KOH	10	1590	1030	14.97	0
167	259.77	6M KOH	20	1590	1030	14.97	0
168	229.67	6M KOH	50	1590	1030	14.97	0
169	197.38	6M KOH	100	1590	1030	14.97	0
170	135.83	6M KOH	200	1590	1030	14.97	0
171	0	1M H_2SO_4	1	0	0	0	0
172	0	1M H_2SO_4	300	0	0	0	0
173	0	1M H_2SO_4	500	0	0	0	0
174	0	1M H_2SO_4	1	0	0	17	15.6
175	0	1M H_2SO_4	300	0	0	17	15.6
176	0	1M H_2SO_4	500	0	0	17	15.6
177	0	1M H_2SO_4	1	0	0	8.5	7.8
178	0	1M H_2SO_4	300	0	0	8.5	7.8
179	0	1M H_2SO_4	500	0	0	8.5	7.8
180	64.47793	1M H_2SO_4	100	1806	113	6.18	0
181	84.81906	1M H_2SO_4	100	1566	116	10.97	0
182	112.0273	1M H_2SO_4	100	1396	160	11.39	0
183	131.7777	1M H_2SO_4	100	1347	84	13.86	0
184	236.0384	1M H_2SO_4	5	1303	2397	7.8	7.7
185	230.6941	1M H_2SO_4	10	1303	2397	7.8	7.7
186	217.1656	1M H_2SO_4	20	1303	2397	7.8	7.7
187	191.6747	1M H_2SO_4	50	1303	2397	7.8	7.7
188	161.1088	1M H_2SO_4	100	1303	2397	7.8	7.7
189	241.4567	1M H_2SO_4	10	1172	350	17.72	6.43
190	219.8804	1M H_2SO_4	20	1172	350	17.72	6.43
191	193.6414	1M H_2SO_4	50	1172	350	17.72	6.43
192	158.8219	1M H_2SO_4	100	1172	350	17.72	6.43

193	108.1825	1M H_2SO_4	200	1172	350	17.72	6.43
194	177.1411	1M H_2SO_4	10	1167	330	9.25	6.9
195	169.7676	1M H_2SO_4	20	1167	330	9.25	6.9
196	155.8553	1M H_2SO_4	50	1167	330	9.25	6.9
197	139.566	1M H_2SO_4	100	1167	330	9.25	6.9
198	116.0936	1M H_2SO_4	200	1167	330	9.25	6.9
199	225.6605	1M H_2SO_4	5	657	680	7.91	4.26
200	200.2923	1M H_2SO_4	10	657	680	7.91	4.26
201	183.7029	1M H_2SO_4	30	657	680	7.91	4.26
202	170.0879	1M H_2SO_4	50	657	680	7.91	4.26
203	145.7881	1M H_2SO_4	100	657	680	7.91	4.26
204	113.5456	1M H_2SO_4	200	657	680	7.91	4.26
205	549.57	1M H_2SO_4	1	3403	498	6.71	1.24
206	457.05	1M H_2SO_4	5	3403	498	6.71	1.24
207	425.67	1M H_2SO_4	10	3403	498	6.71	1.24
208	398.97	1M H_2SO_4	20	3403	498	6.71	1.24
209	383.80	1M H_2SO_4	30	3403	498	6.71	1.24
210	373.11	1M H_2SO_4	40	3403	498	6.71	1.24
211	364.99	1M H_2SO_4	50	3403	498	6.71	1.24
212	350.91	1M H_2SO_4	75	3403	498	6.71	1.24
213	340.23	1M H_2SO_4	100	3403	498	6.71	1.24
214	324.43	1M H_2SO_4	150	3403	498	6.71	1.24
215	312.07	1M H_2SO_4	200	3403	498	6.71	1.24
216	292.45	1M H_2SO_4	300	3403	498	6.71	1.24
217	276.32	1M H_2SO_4	400	3403	498	6.71	1.24
218	262.06	1M H_2SO_4	500	3403	498	6.71	1.24
219	520.29	1M H_2SO_4	1	2708	253	16.76	1.52
220	426.43	1M H_2SO_4	5	2708	253	16.76	1.52
221	394.85	1M H_2SO_4	10	2708	253	16.76	1.52
222	368.50	1M H_2SO_4	20	2708	253	16.76	1.52

223	353.79	1M H_2SO_4	30	2708	253	16.76	1.52
224	343.66	1M H_2SO_4	40	2708	253	16.76	1.52
225	335.86	1M H_2SO_4	50	2708	253	16.76	1.52
226	322.71	1M H_2SO_4	75	2708	253	16.76	1.52
227	312.78	1M H_2SO_4	100	2708	253	16.76	1.52
228	298.44	1M H_2SO_4	150	2708	253	16.76	1.52
229	287.41	1M H_2SO_4	200	2708	253	16.76	1.52
230	270.43	1M H_2SO_4	300	2708	253	16.76	1.52
231	256.94	1M H_2SO_4	400	2708	253	16.76	1.52
232	245.36	1M H_2SO_4	500	2708	253	16.76	1.52
233	557.82	1M H_2SO_4	1	2796	540	17.22	0.83
234	458.60	1M H_2SO_4	5	2796	540	17.22	0.83
235	425.05	1M H_2SO_4	10	2796	540	17.22	0.83
236	396.20	1M H_2SO_4	20	2796	540	17.22	0.83
237	379.57	1M H_2SO_4	30	2796	540	17.22	0.83
238	367.84	1M H_2SO_4	40	2796	540	17.22	0.83
239	358.75	1M H_2SO_4	50	2796	540	17.22	0.83
240	343.08	1M H_2SO_4	75	2796	540	17.22	0.83
241	331.07	1M H_2SO_4	100	2796	540	17.22	0.83
242	313.26	1M H_2SO_4	150	2796	540	17.22	0.83
243	299.34	1M H_2SO_4	200	2796	540	17.22	0.83
244	277.38	1M H_2SO_4	300	2796	540	17.22	0.83
245	259.52	1M H_2SO_4	400	2796	540	17.22	0.83
246	243.92	1M H_2SO_4	500	2796	540	17.22	0.83
247	616.64	1M H_2SO_4	1	3650	826	11.78	1.56
248	506.54	1M H_2SO_4	5	3650	826	11.78	1.56
249	469.49	1M H_2SO_4	10	3650	826	11.78	1.56
250	437.89	1M H_2SO_4	20	3650	826	11.78	1.56
251	419.71	1M H_2SO_4	30	3650	826	11.78	1.56
252	406.83	1M H_2SO_4	40	3650	826	11.78	1.56

253	396.92	1M H_2SO_4	50	3650	826	11.78	1.56
254	379.58	1M H_2SO_4	75	3650	826	11.78	1.56
255	366.18	1M H_2SO_4	100	3650	826	11.78	1.56
256	346.25	1M H_2SO_4	150	3650	826	11.78	1.56
257	330.45	1M H_2SO_4	200	3650	826	11.78	1.56
258	305.49	1M H_2SO_4	300	3650	826	11.78	1.56
259	285.47	1M H_2SO_4	400	3650	826	11.78	1.56
260	268.47	1M H_2SO_4	500	3650	826	11.78	1.56
261	481.92	1M H_2SO_4	1	3144	909	4.46	1.27
262	396.66	1M H_2SO_4	5	3144	909	4.46	1.27
263	371.29	1M H_2SO_4	10	3144	909	4.46	1.27
264	349.30	1M H_2SO_4	20	3144	909	4.46	1.27
265	336.61	1M H_2SO_4	30	3144	909	4.46	1.27
266	327.80	1M H_2SO_4	40	3144	909	4.46	1.27
267	320.93	1M H_2SO_4	50	3144	909	4.46	1.27
268	309.12	1M H_2SO_4	75	3144	909	4.46	1.27
269	300.02	1M H_2SO_4	100	3144	909	4.46	1.27
270	286.64	1M H_2SO_4	150	3144	909	4.46	1.27
271	276.19	1M H_2SO_4	200	3144	909	4.46	1.27
272	259.88	1M H_2SO_4	300	3144	909	4.46	1.27
273	246.73	1M H_2SO_4	400	3144	909	4.46	1.27
274	235.37	1M H_2SO_4	500	3144	909	4.46	1.27
275	554.63	1M H_2SO_4	1	1691	258	16.45	3.31
276	440.96	1M H_2SO_4	5	1691	258	16.45	3.31
277	403.28	1M H_2SO_4	10	1691	258	16.45	3.31
278	362.32	1M H_2SO_4	20	1691	258	16.45	3.31
279	333.81	1M H_2SO_4	30	1691	258	16.45	3.31
280	313.43	1M H_2SO_4	40	1691	258	16.45	3.31
281	296.64	1M H_2SO_4	50	1691	258	16.45	3.31
282	267.53	1M H_2SO_4	75	1691	258	16.45	3.31

283	246.87	1M H_2SO_4	100	1691	258	16.45	3.31
284	218.17	1M H_2SO_4	150	1691	258	16.45	3.31
285	198.38	1M H_2SO_4	200	1691	258	16.45	3.31
286	171.19	1M H_2SO_4	300	1691	258	16.45	3.31
287	152.27	1M H_2SO_4	400	1691	258	16.45	3.31
288	137.40	1M H_2SO_4	500	1691	258	16.45	3.31

Note: 10-25[1]; 26-31[2]; 32-43[3]; 44-60[4]; 61-67[5]; 68-75[6]; 76-99[7]; 100-104[8]; 105-113[9]; 114-119[10]; 120-126[11]; 127-138[12]; 139-148[13]; 149-158[14]; 159-164[15]; 165-170[16];180-183[17];184-188[18]; 189-198[19]; 199-204[20]; 205-288: this work.

Supplementary Note 1

In this work, an artificial neural network (ANN) model is used to predict the performance of N/O co-doped activated carbon electrodes in supercapacitors. All data used in the ANN model is provided in Supplementary Table 1, collected from the literature[1-21]. We also reinforced the ML model with additional experimental data collected in this work. The structural features used in ANN include micropore surface area (S_{micro}) and mesopore surface area (S_{meso}), while the total percentage of nitrogen and oxygen doping is used as the chemical features. In addition, the scan rate is also used to capture the decrease of capacitance at a faster charging/discharging rate. In the training database, the electrochemical performance of N/O co-doped activated carbon electrodes are collected in both 6M KOH and 1M H_2SO_4 electrolytes. In the ANN model, the type of electrolyte is treated as a dummy variable. The percentages of training, validation, and test data are 70%, 15%, and 15%. The artificial neural network (ANN) includes a layer of 7 neurons as a hidden layer with a hyperbolic tangent sigmoid transfer function. The backpropagation employs Bayesian regularization, making the ANN more robust and generalized without overfitting.[22]

Supplementary Note 2

Following the guideline from IUPAC for the assessment of the surface area, a physisorption isotherm is transformed into the 'BET plot', which derives a value of the BET monolayer capacity (Qm). It is customary to apply the BET equation in the linear form:

$$\frac{p/p^\circ}{Q(1-p/p^\circ)} = \frac{1}{Q_m C} + \frac{C-1}{Q_m C}(p/p^\circ)$$

where Q is the specific amount adsorbed at the relative pressure p/p° and Qm is the specific monolayer capacity. Then, the BET area is calculated by adopting an appropriate value of the molecular cross-sectional area. For applying the BET method to microporous materials, a useful procedure (Rouquerol plots) allows one to avoid subjectivity in evaluating the BET monolayer capacity.[24] Here the physisorption isotherm plots in the p/p° range of 0.05 to 0.35 was transformed into the BET surface area plot. According to Rouquerol plots of C-Phl, five plots where the Q(1 – p/p°) continuously increases in the p/p° range of 0.12 to 0.23 was selected for the calculation of BET surface area. The C value is 50, and the correlation coefficient is 0.99997. Thus, the calculation of BET surface area meets the requirements of IUPAC, and the ultrahigh BET surface area of 4476 m^2/g validates our design's rationality for synthesizing hyperporous carbons.

Supplementary Note 3

In Supplementary Figure 17a, the elastic scattering intensity scans summed over Q = 0.3–1.1Å^{-1} are presented for the dry samples and the wet samples. In the figure, the data have been normalized by the mass of the samples. For the dry carbons, the monotonical behavior of the curves indicates that all the scattering species in these materials are either immobilized or perform motions that are not within the instrumental resolution. Here, the very slight decrease in intensity of the elastic scattering as the temperature increases is solely related to the Debye-Waller factor, and the very similar absolute intensities in both the dry samples indicate that these have very comparable numbers of H-rich species. For the wet samples, the higher scattering intensities as caused by the presence of the electrolyte can be contemplated. In Supplementary Figure 17b, the data from the wet samples are reproduced with the datapoint at T = 20 K normalized to unity to allow for the observation of the temperature-dependent changes in the fraction of elastic scattering. In Supplementary Figure 17c, the Q-dependence of the fractions of the elastic scattering intensities in the QENS signals at 300 K is presented and provides information about the portion of the material that does not perform detectable motions, as depicted by the data points at the highest Q-values. As expected, based on the above-described synthesis of the materials and the respective final fractions of carbon in each sample, C-Phl-700 presents a lower percent of elastic scattering, 12%, than C-Phl, 20%.

Turning to the QENS spectra collected at 300K, these are presented in Figure 5b at Q = 0.7 Å^{-1}. In Figure 5b, the QENS spectra are presented as the imaginary part of the dynamical susceptibilities, obtained via the following expression:

$$\chi''(Q,E) \propto \frac{S(Q,E)}{n_B(T,E)} = S(Q,E) * \left[exp\left(\frac{E}{kT}\right) - 1\right] \qquad \text{(S2)}$$

where $n_B(T, E)$ is the temperature Bose factor, k is the Boltzmann constant, T is the temperature, and $S(Q, E)$ is the dynamic scattering function. For the presentation in Supplementary Figure 18a, the signals from the dry samples could also be subtracted from the data from the wet materials to mitigate the influences of elastic scattering at the lowest frequencies. This test has been made and a similar outcome was obtained. With the presentation in Supplementary Figure 18a, visual perception of two distinct components is possible in both samples. That is, relaxations above 10 μeV are detected as well as a component around 1 μeV. In the following lines, the origins of these components will be discussed and in Supplementary Figure 18 b and c, their Q-dependences can be appreciated.

The component around 1 μeV, which is visually different for C-Phl and C-Phl-700, is associated with the elastic scattering, hence with $A_0(Q)$. Indeed, if this component was associated not with elastic signal, but slow dynamics within the materials, as represented by $\Gamma_N(Q)$ in equation (5), discrepancies in the p-factor, discussed in the main manuscript (see Figure 5d), between the two samples would be detected. Since this is not the case, the ca. 1 μeV component must be elastic. Still, the presence of two dynamic processes is indicated by the two transitions in the elastic scattering intensity scans, displayed in Supplementary Figure 17, and fitting $S(Q, E)$ with a single component, either Lorentzian or a stretched function, does not lead to satisfactory results. Therefore, we assume that the relaxation signal above 10 μeV in Supplementary Figure 18a is composed of a slower and a faster component, associated with $\Gamma_N(Q)$ and $\Gamma_B(Q)$ in Equation (5). If this supposition is valid, then the slope at the low-frequency side of the main relaxation must differ significantly from unity, indicating that the dynamic process under analysis does not consist

of a single component. As shown in Supplementary Figure 19, this is, indeed, the case (for completeness, the slopes at the high-frequency side are also shown).

Supplementary Reference

[1] Wu, H. *et al.* The effect of activation technology on the electrochemical performance of calcium carbide skeleton carbon. *J Solid State Electr* **16**, 2941-2947, (2012).

[2] Zhang, J., Gong, L., Sun, K., Jiang, J. & Zhang, X. Preparation of activated carbon from waste Camellia oleifera shell for supercapacitor application. *J Solid State Electr* **16**, 2179-2186, (2012).

[3] Chen, X. Y. *et al.* Nitrogen-doped porous carbon for supercapacitor with long-term electrochemical stability. *J. Power Sources* **230**, 50-58, (2013).

[4] Wang, Y., Yang, R., Wei, Y., Zhao, Z. & Li, M. Preparation of novel pigskin-derived carbon sheets and their low-temperature activation-induced high capacitive performance. *Rsc Adv* **4**, 45318-45324, (2014).

[5] Li, Y.-T., Pi, Y.-T., Lu, L.-M., Xu, S.-H. & Ren, T.-Z. Hierarchical porous active carbon from fallen leaves by synergy of K2CO3 and their supercapacitor performance. *J Power Sources* **299**, 519-528, (2015).

[6] Tan, J., Chen, H., Gao, Y. & Li, H. Nitrogen-doped porous carbon derived from citric acid and urea with outstanding supercapacitance performance. *Electrochim Acta* **178**, 144-152, (2015).

[7] Jiang, L., Sheng, L., Chen, X., Wei, T. & Fan, Z. Construction of nitrogen-doped porous carbon buildings using interconnected ultra-small carbon nanosheets for ultra-high rate supercapacitors. *J Mater Chem A* **4**, 11388-11396, (2016).

[8] Hao, X. *et al.* Bacterial-cellulose-derived interconnected meso-microporous carbon nanofiber networks as binder-free electrodes for high-performance supercapacitors. *J Power Sources* **352**, 34-41, (2017).

[9] Yang, W. *et al.* Template-free synthesis of ultrathin porous carbon shell with excellent conductivity for high-rate supercapacitors. *Carbon* **111**, 419-427, (2017).

[10] Zhang, D. *et al.* Scalable synthesis of hierarchical macropore-rich activated carbon microspheres assembled by carbon nanoparticles for high rate performance supercapacitors. *J Power Sources* **342**, 363-370, (2017).

[11] Liu, W. *et al.* Nitrogen-Doped Hierarchical Porous Carbon from Wheat Straw for Supercapacitors. *Acs Sustain Chem Eng* **6**, 11595-11605, (2018).

[12] Song, Z. *et al.* Nitrogen-Enriched Hollow Porous Carbon Nanospheres with Tailored Morphology and Microstructure for All-Solid-State Symmetric Supercapacitors. *Acs Appl Energ Mater* **1**, 4293-4303, (2018).

[13] Zhu, D. *et al.* A general strategy to synthesize high-level N-doped porous carbons via Schiff-base chemistry for supercapacitors. *J Mater Chem A* **6**, 12334-12343, (2018).

[14] Song, Z. *et al.* Synergistic design of a N, O co-doped honeycomb carbon electrode and an ionogel electrolyte enabling all-solid-state supercapacitors with an ultrahigh energy density. *J Mater Chem A* **7**, 816-826, (2019).

[15] Xue, D. *et al.* Template-Free, Self-Doped Approach to Porous Carbon Spheres with High N/O Contents for High-Performance Supercapacitors. *Acs Sustain Chem Eng* **7**, 7024-7034, (2019).

[16] Ma, C. *et al.* Sustainable recycling of waste polystyrene into hierarchical porous carbon nanosheets with potential applications in supercapacitors. *Nanotechnology* **31**, 035402, (2020).

[17] Tang, Q. *et al.* Integrating Effect of Surface Modification of Microporous Carbon by Phosphorus/Oxygen as well as the Redox Additive of p‐Aminophenol for High‐Performance Supercapacitors. *Advanced Materials Interfaces* **7**, (2020).

[18] Zhou, X. *et al.* Biomass based nitrogen-doped structure-tunable versatile porous carbon materials. *J Mater Chem A* **5**, 12958-12968, (2017).

[19] Liu, Y. *et al.* N–O–S Co-doped Hierarchical Porous Carbons Derived from Calcium Lignosulfonate for High-Performance Supercapacitors. *Energy & Fuels* **34**, 3909-3922, (2020).

[20] Mu, J. *et al.* Fishbone-derived N-doped hierarchical porous carbon as an electrode material for supercapacitor. *Journal of Alloys and Compounds* **832**, (2020).

[21] Chen, X. Y., Chen, C., Zhang, Z. J., Xie, D. H. & Deng, X. Nitrogen-Doped Porous Carbon Prepared from Urea Formaldehyde Resins by Template Carbonization Method for Supercapacitors. *Ind Eng Chem Res* **52**, 10181-10188, (2013).

[22] Burden, F. & Winkler, D. Bayesian regularization of neural networks. *Methods Mol Biol* **458**, 25-44, (2008).
